# Casein kinase TbCK1.2 regulates division of kinetoplast DNA, and movement of basal bodies in the African trypanosome

**DOI:** 10.1371/journal.pone.0249908

**Published:** 2021-04-16

**Authors:** Catherine Sullenberger, Benjamin Hoffman, Justin Wiedeman, Gaurav Kumar, Kojo Mensa-Wilmot

**Affiliations:** 1 Department of Cellular Biology, University of Georgia, Athens, GA, United States of America; 2 Department of Molecular and Cellular Biology, Kennesaw State University, Kennesaw, GA, United States of America; 3 Center for Tropical and Emerging Global Diseases, University of Georgia, Athens, GA, United States of America; Louisiana State University, UNITED STATES

## Abstract

The single mitochondrial nucleoid (kinetoplast) of *Trypanosoma brucei* is found proximal to a basal body (mature (mBB)/probasal body (pBB) pair). Kinetoplast inheritance requires synthesis of, and scission of kinetoplast DNA (kDNA) generating two kinetoplasts that segregate with basal bodies into daughter cells. Molecular details of kinetoplast scission and the extent to which basal body separation influences the process are unavailable. To address this topic, we followed basal body movements in bloodstream trypanosomes following depletion of protein kinase TbCK1.2 which promotes kinetoplast division. In control cells we found that pBBs are positioned 0.4 um from mBBs in G1, and they mature after separating from mBBs by at least 0.8 um: mBB separation reaches ~2.2 um. These data indicate that current models of basal body biogenesis in which pBBs mature in close proximity to mBBs may need to be revisited. Knockdown of TbCK1.2 produced trypanosomes containing one kinetoplast and two nuclei (1K2N), increased the percentage of cells with uncleaved kDNA 400%, decreased mBB spacing by 15%, and inhibited cytokinesis 300%. We conclude that (a) separation of mBBs beyond a threshold of 1.8 um correlates with division of kDNA, and (b) TbCK1.2 regulates kDNA scission. We propose a Kinetoplast Division Factor hypothesis that integrates these data into a pathway for biogenesis of two daughter mitochondrial nucleoids.

## Introduction

The single-cell eukaryote *Trypanosoma brucei* causes human African trypanosomiasis (HAT) in some regions of sub-Saharan Africa. The trypanosome mitochondrial genome, comprised of catenated double-stranded DNAs, is organized as a single nucleoid termed “kinetoplast” [1–3]. Loss of kinetoplast DNA (kDNA) disrupts mitochondrial membrane potential in stumpy form bloodstream trypanosomes [4,5] and interferes with development of the parasite in the tsetse fly vector, breaking the vector-to-mammal transmission cycle that is needed to spread the disease [6].

Kinetoplast DNA (kDNA) is unique in biology because it is comprised of two classes of circular double-stranded DNAs, minicircles and maxicircles, that are catenated into a network (reviewed in [2,7,8]). Each minicircle is interlocked with three neighbors [9], and the maxicircles are threaded into the honeycomb arrangement of minicircles to form kDNA. A kDNA network is divided in two [10] by unknown enzymes (reviewed in [11]) so that progeny networks can be sorted into two daughter trypanosomes at cytokinesis (reviewed in [12]).

The cycle of kinetoplast biogenesis has five steps, minimally. To assist a reader in following our narrative, we define terminology used in this manuscript since the same words are used to describe different events throughout the literature. “Synthesis” of kDNA is the incorporation of nucleotides into a kinetoplast. “Selection of Scission Site” describes the positioning of the cleavage site on kDNA. “Scission/Cleavage” involves resolution of a kDNA into two networks. “Separation” refers to initial movement apart of cleaved kinetoplasts. “Sorting” is the distribution of kinetoplasts into daughter trypanosomes at cytokinesis. “Division” combines “scission” and “initial separation” of kinetoplasts. “Segregation” of kinetoplasts has no precise molecular definition in the field [10–12]: We define it as “post-division movements” leading to inheritance of kDNA (reviewed in [12]).

Division of kDNA precedes, and is essential for, segregation of kinetoplasts (reviewed in [11]). Little is known about how replicated kDNA is equally divided in two kinetoplasts, although excellent progress has been made in understanding how kDNA is synthesized (reviewed in [2]). It is envisioned to require several molecular activities: identification of precise scission sites on kDNA, directed (vectorial) scission of kDNA without decatenation of the minicircle and maxicircle constituents, and coordination with the cell cycle [11]. Proteins that mediate any of the molecular events required for division of kinetoplasts have yet to be identified (reviewed in [11]).

Basal bodies (centrioles) are localized near kinetoplasts; they facilitate assembly of flagellar axonemes [13] and interact with the cytoskeleton as well as membranous structures [14]. A mature basal body has three sections: (i) a proximal region organized by nine triplet microtubules, (ii) a distally positioned transition zone characterized by nine doublet microtubules, and (iii) and a distal basal plate which caps the end of the basal body linking it the central microtubule pair of the flagellar axoneme [14]. In G1 a mature basal body and pro-basal body are present in *T*. *brucei*. A pro-basal body has triplet microtubules, but it lacks a transition zone and an axoneme. Molecular markers of different segments of basal bodies are known. Cartwheel protein TbSas6p is detected at the base of the triplet microtubules of pro-basal bodies and mature basal bodies [15,16] and several transition zone proteins, including TbRP2 [17], have been identified [18].

Maturation of pro-basal bodies involves extension of the transition zone, similar to the growth of pro-centrioles during their conversion to basal bodies in human cells [19]. Morphological studies indicate that a new mature basal body rotates around the old basal body in *T*. *brucei* [20]. However, proteins required for maturation of basal bodies have not been identified in *T*. *brucei*. Consequently, unlike pro-centriole maturation in human cells (reviewed in [21]), the proteins that regulate pro-basal body disengagement and maturation are yet to be determined in *T*. *brucei*.

Duplication of basal bodies and kinetoplasts is coordinated with the trypanosome division cycle. In G1 bloodstream trypanosomes have one kinetoplast (K), one nucleus (N) (1K1N), and one basal body. kDNA is synthesized, and the basal body duplicates in S-phase. Kinetoplast division is detected in G2. Mitosis in 2K1N trypanosomes produces 2K2N cells that after cytokinesis yield two 1K1N cells [22,23].

Protein kinase regulation of mitochondrial nucleoid division or separation has not been described in any biological system. In *T*. *brucei*, knockdown of a casein kinase TbCK1.2 inhibits kinetoplast division [24,25], while mitosis progresses normally. Consequently, a population of “mutant” trypanosomes with a single kinetoplast and two nuclei (1K2N) arises [24]. We hypothesized that loss of TbCK1.2 disrupted kinetoplast division by preventing one or more of six processes; (a) synthesis of kDNA, (b) scission of kinetoplasts, (c) separation of cleaved kDNAs, (d) basal body duplication, (e) movement of basal bodies, or (f) flagellum nucleation.

We find that kDNA synthesis occurs in 1K2N trypanosomes. Compared to control cells, 1K2N cells separate basal bodies to normal overall extents, although the distribution of inter-basal body distances contracted in them. There was a 4-fold increase in the fraction of uncleaved kDNA in the population, indicating that TbCK1.2 facilitates kDNA scission. These data document failure of kDNA scission even after separation of basal bodies, providing genetic evidence that separation of basal bodies is not sufficient to divide kinetoplasts. TbCK1.2 is a founding member of a group of proteins that are required for division of kinetoplasts (*i*.*e*., kinetoplast division factors) (discussed in [11]). We propose a “kinetoplast division factor” (KDF) hypothesis to (i) explain the uncoupling of basal body separation from division of kDNA, and (ii) integrate all available new data into a working hypothesis for division and inheritance of the mitochondrial genome in a trypanosome.

## Results

### TbCK1.2 regulates division of kDNA in *T*. *brucei*

The mitochondrial genome of the African trypanosome is organized as one nucleoid (kinetoplast) [2]. To ensure inheritance of this genome during cell division, kinetoplast DNA (kDNA) synthesis, division (*i*.*e*., scission and initial separation), and inheritance are coordinated with the cell cycle (S1 Fig). Division of kDNA is a poorly understood process, although many genes involved in post-division segregation have been identified (reviewed in [3,11]).

**Fig 1:**
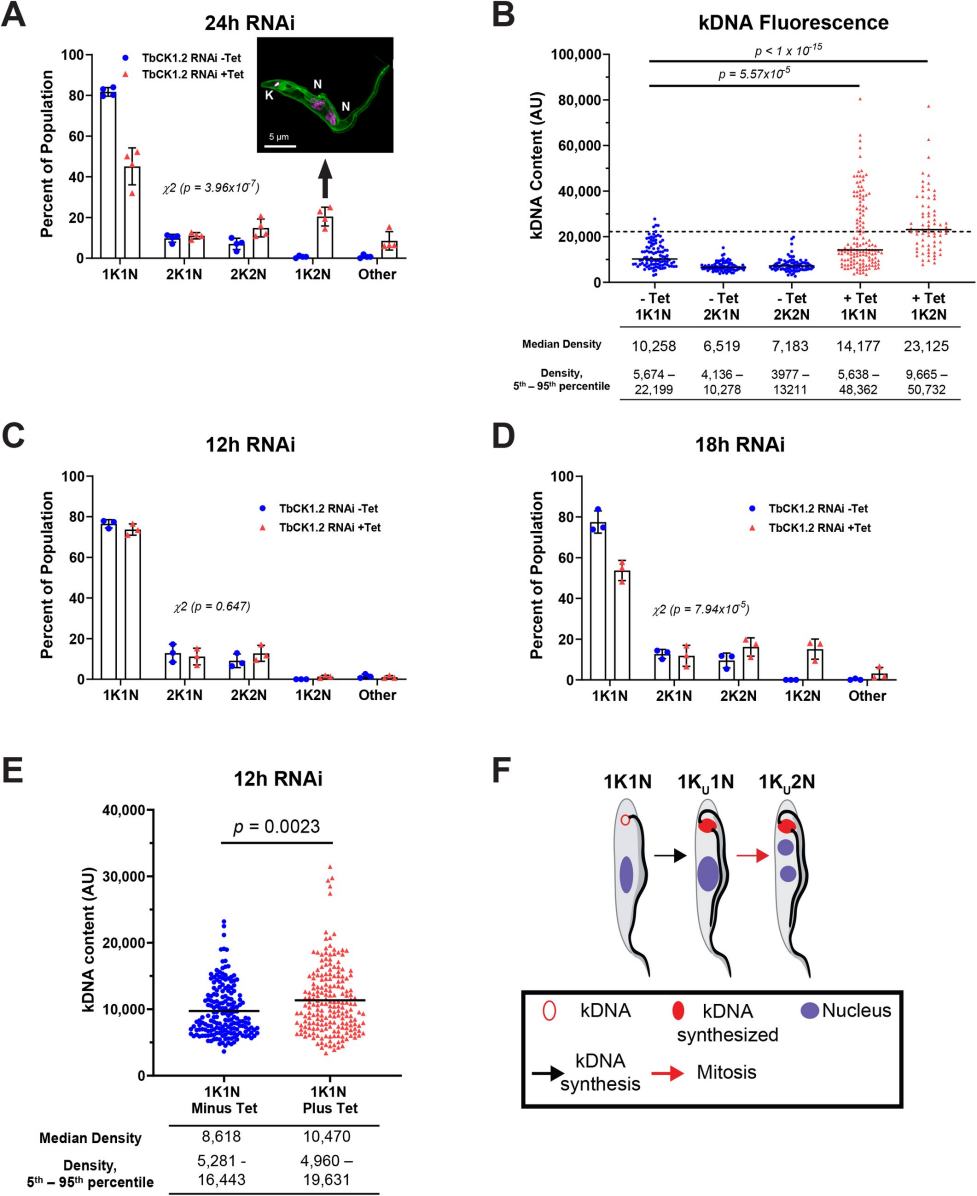
Knockdown of TbCK1.2 blocks division of kinetoplasts: Time-course of the effect of RNAi against TbCK1.2 on kinetoplasts and nuclei. **(A)** Effects on kinetoplast duplication were assessed by enumeration of the number of kinetoplasts (K) and nuclei (N) per trypanosome in cells cultured in the absence or presence of tetracycline (1 μg/mL, 24 h) (“Other” indicates cells with >2 or <1 K/N). Error bars represent standard deviation of four independent biological experiments (n = 110-268/experimental sample). A ***χ***^2^ test was used to determine whether the difference in distribution of kinetoplasts and nuclei was statistically significant after knockdown of TbCK1.2 (p = 3.96 x 10^−7^). Inset: SR-SIM example image of a 1K2N trypanosome following 24 h of RNAi against TbCK1.2. Cell membranes were labeled with mCLING and DNA was detected with DAPI. **(B)** Effect of knockdown of TbCK1.2 on kinetoplast DNA (kDNA) content. ImageJ was used to measure the fluorescence intensity of individual DAPI-stained kDNA in trypanosomes with one or two kinetoplasts in control (- Tet) or one kinetoplast in TbCK1.2 RNAi (+ Tet) cells. Scatter dot plot relates kDNA fluorescence intensities measured in different trypanosome cell types. The Mann-Whitney U test was used to compare the distribution of fluorescence intensity of DAPI-stained kDNA between -Tet 1K1N and +Tet 1K1N or 1K2N trypanosomes (p = 5.6 x 10^−5^, and p = < 10^−15^, respectively). The 95^th^ percentile of the -Tet 1K1N kDNA content is indicated by the horizontal dotted line. Descriptive statistics corresponding to each sample are aligned beneath the graphs. The effect of TbCK1.2 RNAi on kinetoplast duplication was assessed by enumeration of the number of kinetoplasts (K) and nuclei (N) per trypanosome in cells cultured in the absence or presence of tetracycline (1 μg/mL) for 12 h **(C)** or 18 h **(D)**. (“Other” indicates cells with >2 or <1 K/N). Error bars represent standard deviation of three independent biological experiments (n = >100/experiment). A ***χ***^2^ test was used to determine whether the difference in distribution of kinetoplasts and nuclei was statistically significant after knockdown of TbCK1.2 for 12 h (p = 0.647) or 18 h (p = 7.94 x 10^−5^). (**E**) ImageJ was used to measure DAPI intensity of kDNA fluorescence in trypanosomes with one kinetoplast following 12 h RNAi in control (-Tet) or TbCK1.2 RNAi (+Tet) cells. Violin plot shows the distribution of kDNA fluorescence intensities. A Mann-Whitney U test was used to compare the median fluorescence intensity of DAPI-stained kinetoplasts between -Tet 1K1N (n = 183) and +Tet 1K1N (n = 198) trypanosomes (p = 0.0023). **(*F*)** Cartoon explaining the likely origin of 1K2N trypanosomes from 1K1N cells. Kinetoplast DNA is synthesized in S-phase, forming a cell with an undivided kinetoplast and one nucleus (1K_U_1N) at 12 h. At 18 h of TbCK1.2 knockdown, cells containing two nuclei and one undivided kinetoplast (1K_U_2N) are detected in the population.

Division of kDNA is hampered after knockdown of a casein kinase TbCK1.2 [24] (Fig 1A). To pinpoint the step where TbCK1.2 contributes to division of the kinetoplast, we produced a tetracycline-inducible TbCK1.2 RNAi line [25] in which one allele of the protein was tagged endogenously with a V5 epitope at the N-terminus (V5-TbCK1 RNAi line). Knockdown of TbCK1.2 reduced the level of V5-TbCK1.2 protein by 60% (S2A, S2B, S2D and S2E Fig), and arrested proliferation of trypanosomes within 16 h (S2C Fig). During a normal division cycle, the kinetoplast (K) is divided before mitosis producing cells with two kinetoplasts and one nucleus (2K1N trypanosomes). After knockdown of TbCK1.2 for 24 h a new population of cells with one kinetoplast and two nuclei (1K2N) emerged (Fig 1A). Thus, TbCK1.2 is important for division of the kinetoplast but is not required for mitosis.

The percentage of 1K1N cells was reduced after knockdown of TbCK1.2 (Fig 1A). Compared to the uninduced control, the difference in distribution of kinetoplasts and nuclei per trypanosome was statistically significant (*p* = 3.96 x 10^−7^; ***χ***^2^) after knockdown of TbCK1.2.

Trypanosomes classified as “Other” were present in the population (Fig 1A). In a time-course study, “other cells” are not detected in significant proportions at 12 h (above that in a regular population) after knockdown of TbCK1.2 (Fig 1C). At 18 h, “other” are comprised of 0K1N (1.1%), 1K>2N (0.2%), >2K1N (0.4%), 2K3N (0.2%), 3K2N (0.6%), and >4K>2N (0.4%). None of these subgroups constitutes 5% of the total population, so we do not advertise them as a significant “phenotype” worthy of tracking independently.

### TbCK1.2 is important for cytokinesis

Typically, about 10% of a bloodstream trypanosome population has two nuclei and two kinetoplasts (2K2N), the pre-cytokinesis stage in cell division. After knockdown of TbCK1.2 approximately 30% of cells have two nuclei (counting 1K2N and 2K2N trypanosomes) at 18 h (Fig 1D), and that fraction holds steady at 24 h post-RNAi (Fig 1A). We infer that knockdown of TbCK1.2 for 18 h and beyond leads to failure of cytokinesis (Fig 1A).

### kDNA is replicated after knockdown of TbCK1.2

We examined a hypothesis that inability to duplicate the kinetoplast was the result of failure of kDNA synthesis, i.e., there was not sufficient mitochondrial DNA to partition between two kinetoplasts. Towards this goal, kDNA content of 1K2N trypanosomes was compared to that of kinetoplasts in uninduced (*i*.*e*., for TbCK1.2 knockdown) control cells. Since kDNA synthesis normally occurs in 1K1N trypanosomes before division of the kinetoplast [23,26], 1K1N trypanosomes contain between one-to-two equivalents of kDNA. Division of replicated kDNA yields trypanosomes with two kinetoplasts (2K1N and 2K2N), in which each kinetoplast contains one equivalent of kDNA.

We observed an increase in kDNA content in 1K1N and 1K2N trypanosomes after knockdown of TbCK1.2 for 24 h (Fig 1B). In control cells, the median DAPI fluorescence intensity of kDNA in 1K1N cells (1 x 10^4^ arbitrary units (A.U.)) is approximately twice that in 2K1N trypanosomes (6.5 x 10^3^ A.U.) (Fig 1B). This data is expected since 2K1N are not synthesizing kDNA whereas a fraction of the 1K1N cells is in S-phase. For comparison, kDNA intensity above the 95^th^ percentile of control 1K1N cells (see horizontal dotted line in Fig 1B) is considered “over-replicated”.

After knockdown of TbCK1.2, the median DAPI fluorescence of kDNA (2.6 x 10^4^ A.U.) of 1K2N cells was twice that of control 1K1N cells (*p* = < 10^−15^; Mann-Whitney U Test (Fig 1B). Fifty-five percent of 1K2N kinetoplasts have over-replicated kDNA (as defined above). In 1K1N cells, which comprise 50% of the total population after knockdown of TbCK1.2 (Fig 1A), 33.6% (*i*.*e*., 17% of total population) contain over-replicated kDNA (Fig 1B) (*p* = 5.6x10^-5^; Mann-Whitney U Test). Therefore, 28% of the total population of trypanosomes have over-replicated kDNA; this fraction is obtained by adding two groups of kinetoplasts with over-replicated kDNA: 11% from 1K2N (20% of the total population of which 55% have over-replicated kDNA) (Fig 1A) and 17% from 1K1N (Fig 1B). We conclude that failure of kinetoplast division after knockdown of TbCK1.2 is not a result of failed synthesis of kDNA.

After a 12-h knockdown of TbCK1.2 there was no difference in the kinetoplast/nucleus profiles of control and experimental trypanosomes; neither group contained 1K2N cells (Fig 1C). This observation presented an opportunity to determine whether over-replication of kDNA occurred in 1K1N cells or was restricted to 1K2N trypanosomes, given that kinetoplasts normally divide in 1K1N trypanosomes prior to mitosis [23,27]. We hypothesized that cells with over-replicated kDNA were present in the 1K1N population prior to emergence of 1K2N. To test this concept, we analyzed kDNA content of 1K1N cells at 12-h in control and knockdown cells. Kinetoplasts in 1K1N cells after 12-h RNAi contained more kDNA than control cells (Fig 1E); median fluorescence increased from 8,618 to 10,470 (A.U.), and the difference in distribution of kDNA content was statistically significant (p = 0.0023, Mann-Whitney U-test). Furthermore, fourteen percent of the kinetoplasts in knockdown cells contained more kDNA than the 95^th^ percentile of the control population (Fig 1B). Thus, over-replication of kDNA was detectable in 1K1N trypanosomes before nuclear division. These data are consistent with a model in which 1K1N trypanosomes with over-replicated kDNA (detected at 12-h) convert, after nuclear division, to 1K2N cells observed 18 h after knockdown of TbCK1.2 (Fig 1F).

At the 18-h timepoint there was a decrease in the proportion of 1K1N cells, and 1K2N trypanosomes appeared in the population. Differences in the distribution of cell types was statistically significant (*p* = 7.94 x 10^−5^) (Fig 1D). We conclude that it takes more than 12 h of TbCK1.2 knockdown to produce 1K2N cells.

### Scission of kDNA is inhibited after knockdown of TbCK1.2

**Fig 2:**
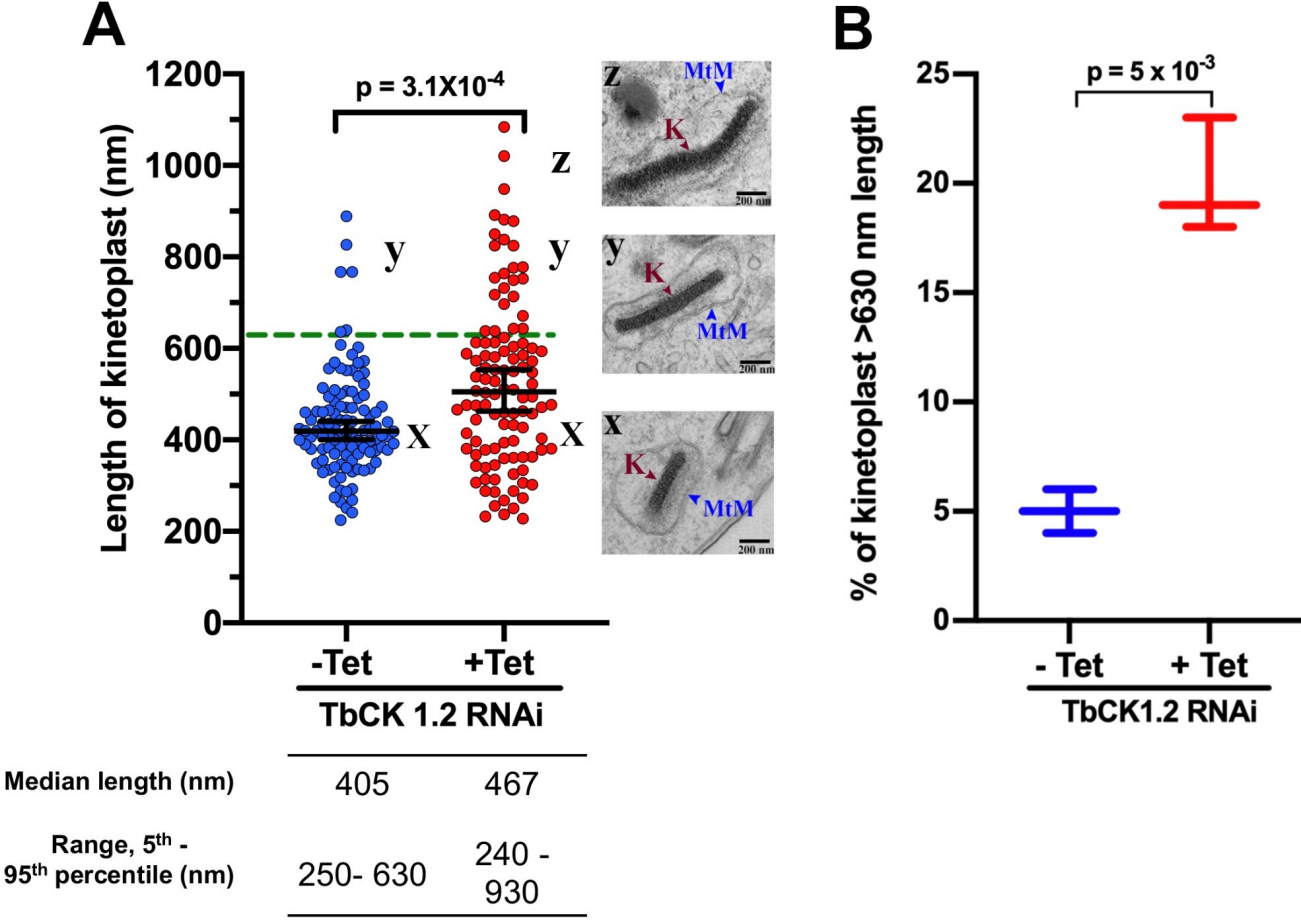
TbCK1.2 knockdown prevents scission of kDNA. Knockdown of TbCK1.2 was induced with tetracycline (1 μg/mL for 24 h). ***(A)*** Scatter dot plot of kinetoplast length in uninduced (-Tet) and induced (+Tet) RNAi TbCK1.2 population. TEM images show kinetoplasts of different lengths (x ≈ 400 nm, y ≈ 800 and z ≈ 1000 nm) in uninduced and induced TbCK1.2 RNAi cells. The 95^th^ percentile of kinetoplast length in control cells (uninduced) is indicated by horizontal dotted line (n = 216 (108, -Tet; 108, +Tet)). K, Kinetoplast; MtM, Mitochondrial membrane; TEM, Transmission Electron Microscopy. *P*-value was calculated using the Mann-Whitney U test. ***(B)*** Percentage of kinetoplasts longer than 630 nm in uninduced and induced populations of TbCK1.2 RNAi cells. Data was analyzed from three replicates, n = 632 (200, first replicate; 216, second and third replicates), P-value was calculated using an Unpaired Student’s t-test.

Since DNA synthesis occurred in kinetoplasts of 1K2N trypanosomes (Fig 1B), we reasoned that kDNA was either uncleaved or had divided but failed to separate by more than 250 nm after scission, resulting in their detection as one kinetoplast by fluorescence microscopy, because of the resolution limit of light [28]. To determine which of these theories was correct we used transmission electron microscopy (TEM) to measure lengths of 632 randomly selected kinetoplasts in multiple fields from 20 ultrathin sections in three independent TEM experiments, a representative of which is presented in Fig 2A (see S3A Fig for electron microscopy images of kinetoplasts at different stages of division and separation).

For control trypanosomes *(i*.*e*., uninduced RNAi line for TbCK1.2), the median length of kinetoplasts was 405 nm (and the 5^th^-to-95^th^ percentile range was 250–630 nm) (Fig 2A). After knockdown of TbCK1.2, the median kinetoplast length increased to 467 nm (the 5^th^-to-95^th^ percentile range was 240–930 nm) (Fig 2A) (*p* = 3.1 x 10^−4^, Mann Whitney U test). Using the 95^th^ percentile length of controls as the limit of normal length (630 nm), we found that 19% of kDNA exceeded this length after knockdown of TbCK1.2 (Fig 2B), representing a four-fold increase in the proportion of uncleaved kDNA (*p* = 5 x 10^−3^, Unpaired Student’s t test). Widths of kinetoplasts were unchanged after knockdown of TbCK1.2 (S3B Fig). These data indicate that knockdown of TbCK1.2 prevents scission of kDNA.

An alternative explanation for these data is that knockdown of TbCK1.2 causes elongation of all kinetoplasts. This possibility is not supported by our data, because the entire distribution of kinetoplast lengths did not shift up after knockdown of TbCK1.2 (Fig 2A).

### Duplication of pro-basal bodies, and kinetics of their separation from mature basal bodies

Basal body (centriole) separation is proposed as a mechanism for segregation of kinetoplasts [1,23,29]. As employed in the literature, “segregation” of kDNA includes the process of dividing kDNA in two (S1 Fig), as well as the post-division movements of kinetoplasts [10,12,30]. For this reason, we investigated a possibility that failed kinetoplast division in 1K2N trypanosomes was caused by inability to duplicate or separate basal bodies.

**Fig 3:**
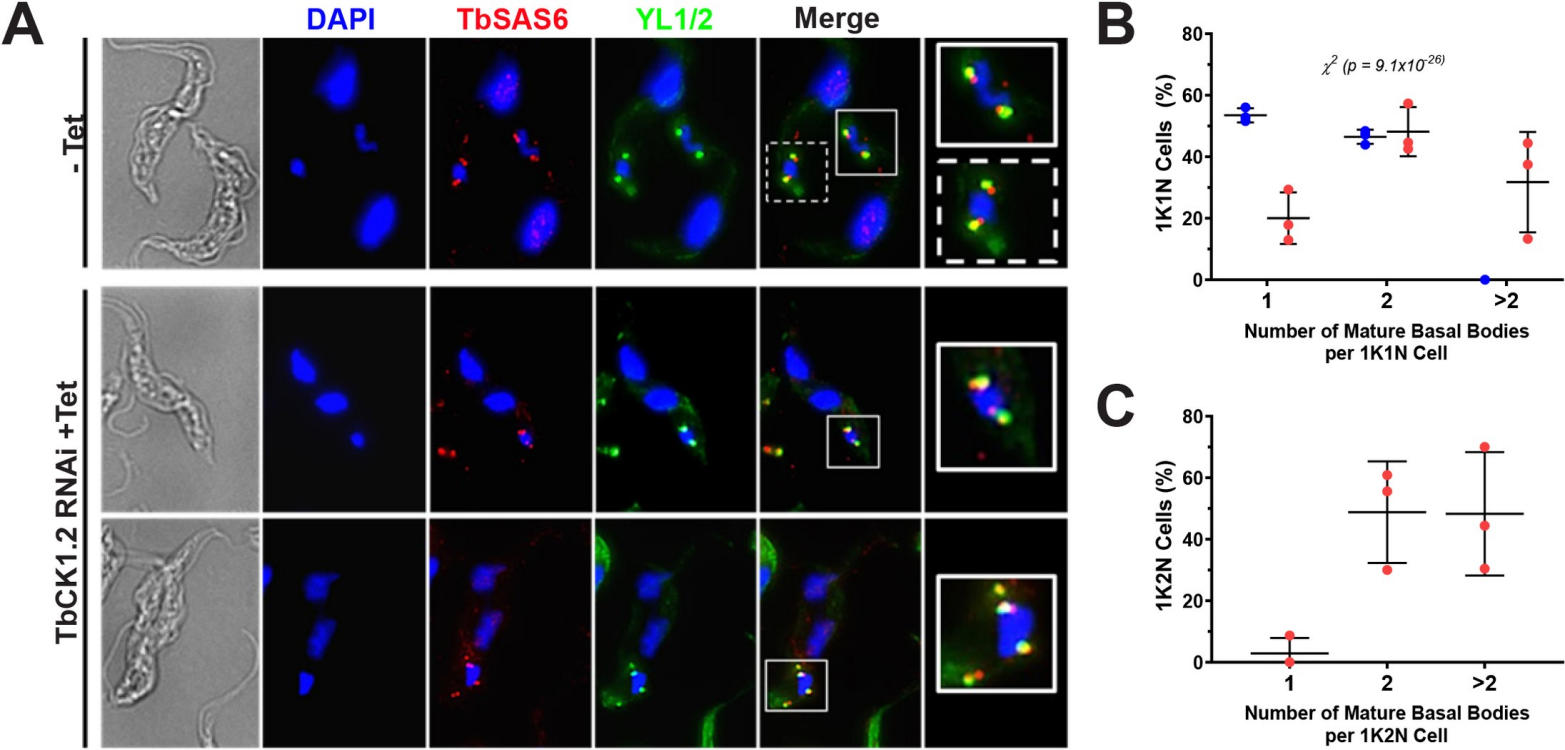
Basal bodies are duplicated after knockdown of TbCK1.2. Knockdown of TbCK1.2 was induced with tetracycline (1 μg/mL for 24 h) and the effect on basal body duplication was determined using antibodies against TbRP2 (YL1/2, mature basal bodies) or TbSAS6 (detects mature and probasal bodies). DAPI was used to stain DNA. ***(A)*** Images represent 1K1N trypanosomes from control cells (-Tet) or 1K2N cells from TbCK1.2-depleted cells (+Tet) with two (2BB) or more (>2BB) basal bodies. Boxed regions are enlarged in the final panel. The average percentage of cell types with the indicated number of mature basal bodies (mBB, YL1/2+) is shown for 1K1N ***(B)*** or 1K2N trypanosomes ***(C)*** from three independent experiments (n = 108-128/experiment). Blue and red symbols indicate control and RNAi treated samples, respectively. Error bars denote standard deviation. The distribution of mBBs in 1K1N trypanosomes after knockdown of TbCK1.2 was compared to control cells (-Tet) using a ***χ***2 test (1K1N *p* = 9.1 x 10^−26^). (A ***χ***2 test could not performed for the 1K2N population because those cells were undetectable (< 1%) in the uninduced population).

Control 1K1N trypanosomes had one or two basal bodies (Fig 3A) [22,23]. After knockdown of TbCK1.2, most 1K1N and 1K2N trypanosomes had two (or more) basal bodies (Fig 3A and 3C). Hence, impaired kinetoplast division is not the result of failed duplication of basal bodies.

Interestingly, 30% of 1K1N cells (15% of the total cell population) (Fig 3B), and 50% of 1K2N trypanosomes (10% of the total population of trypanosomes) (Fig 3C) had more than two basal bodies. Thus TbCK1.2 regulates copy number of basal bodies, in addition to separation of the organelle (see next section, and also Discussion).

Centrioles (basal bodies) are typically found as a mother and daughter pair, each of which has a mature centriole (basal body) and a procentriole (pro-basal body) [31,32]. During cell proliferation, procentrioles disengage, separate from mature centrioles, and mature by acquiring other proteins and structures (*e*.*g*. appendages) [33,34]. In *T*. *brucei*, separation of pro-basal bodies (pBBs) from mature basal bodies (mBBs) has not been studied. Addressing this topic experimentally in *T*. *brucei* calls for a system in which duplication and maturation of pro-basal bodies may be controlled experimentally.

**Fig 4:**
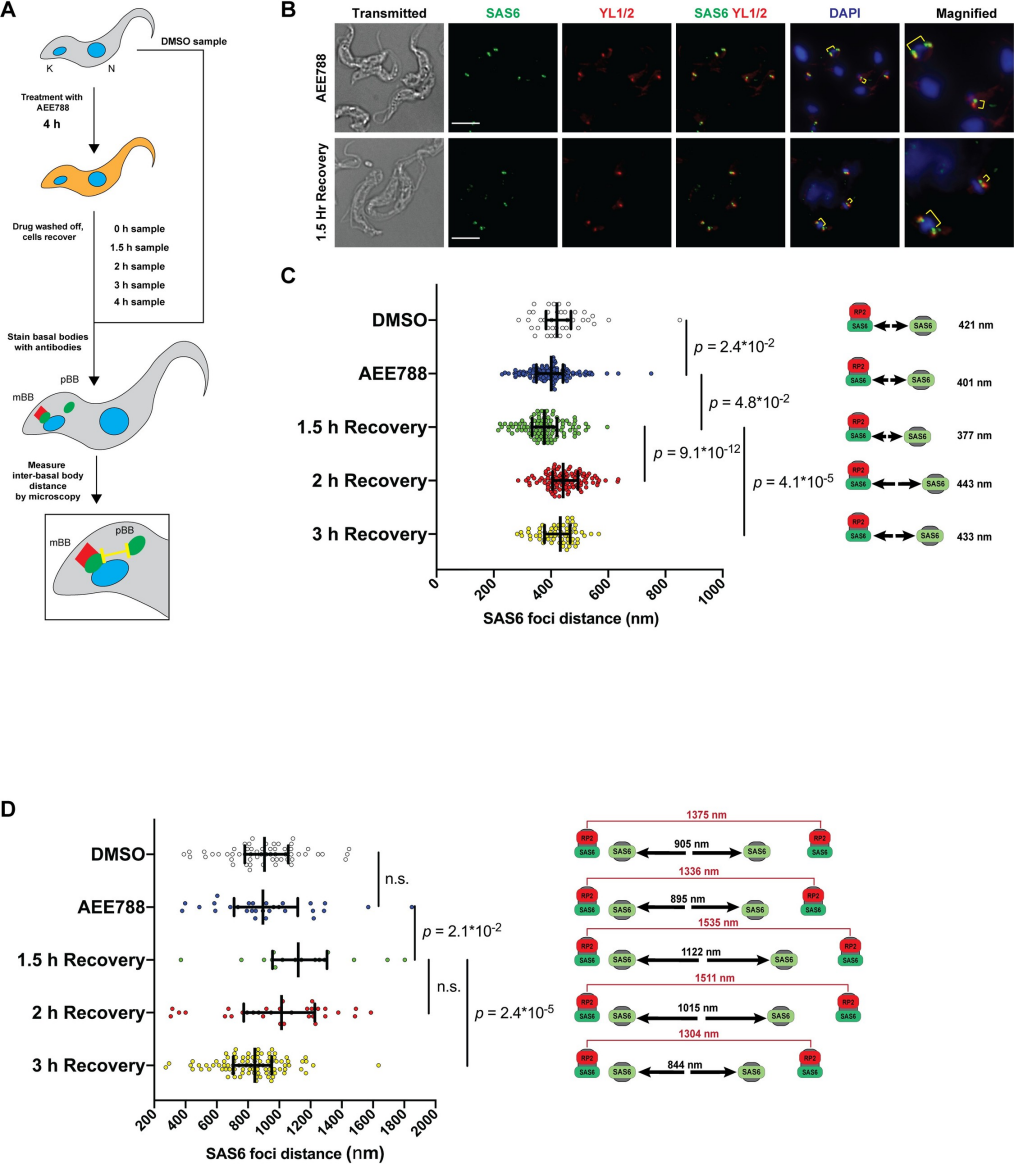
Evidence of probasal body movement in *T*. *brucei*. (*A*) Schematic of protocol used in the study. Trypanosomes were treated with AEE788 (5 μM) or DMSO (control) for 4 hours, released from drug pressure, and allowed to recover for 1.5, 2, or 3 hours. Antibodies against TbRP2 (YL1/2) and TbSAS6 were used to identify basal bodies via immunofluorescence microscopy. ImageJ was used to measure inter-basal body distances by tracking separation between centers of TbSAS6 puncta. *(B)* Representative images of cells from AEE788-treated group, and cells allowed to recover from drug for 1.5 h. Separation between basal bodies is highlighted in yellow. Scale bar = 5 μm. *(C)* Plot shows distances between pro-basal bodies (TbSAS6 positive) and mature basal bodies (TbRP2/TbSAS6 positive) in cells with one mature basal body (mBB). Bars on graph indicate median and inter-quartile range. Numbers to the right indicate median inter-basal body distances for each group. Trypanosomes were drawn from a single experiment. Cells analyzed = 41 (DMSO), 131 (AEE788), 99 (1.5 h recovery), 106 (2 h recovery), 62 (3 h recovery). Inter-basal body distances were compared between groups with a Mann-Whitney U test. The difference in distribution of inter-basal body distances between DMSO treated group and AEE788 treated group was statistically significant (*p* = 2.4*10^−2^). The difference in inter-basal body distances between the group harvested immediately after AEE788 treatment and the population given 1.5 h to recover was statistically significant (*p* = 4.8*10^−2^). The difference between the 1.5 h recovery and 2 h recovery groups was highly statistically significant (*p* = 9.1*10^−12^). The difference in inter-basal body distance between the 2 h and 3 h group was statistically significant (*p* = 4.1*10^−5^). *(D)* Distances between pro-basal bodies (pairs of TbSAS6-positive foci) in cells with two mature basal bodies (mBB) are plotted. Bars on graph show median and inter-quartile range. Numbers to the right in black indicate median distances between a mature basal body and a pro-basal body for each group. Numbers in red denote distances (median) between pairs of mature basal bodies for each group. Cells analyzed = 59 (DMSO), 29 (AEE788), 16 (1.5 h recovery), 32 (2 h recovery), 92 (3 h recovery). Inter-basal body distances were compared between groups with a Mann-Whitney U test. The difference in distribution of inter-basal body distances between DMSO treated group and AEE788 treated group was not statistically significant. The difference in distribution of inter-basal body distances between the AEE788 treatment group and the group at 1.5 h recovery was statistically significant (*p* = 2.1*10^−2^). The difference in inter-basal body distances in 1.5 h recovery and 2 h recovery groups was not statistically significant. The difference in inter-basal body distance between the 2 h and 3 h group was statistically significant (*p* = 2.4*10^−5^). Distances between mature basal bodies (pairs of TbRP2-positive foci) in the same cells are listed to the right in red.

The small-molecule AEE788 [35] may be used to block biogenesis of mature basal bodies in bloodstream *T*. *brucei* [23]. Washing off AEE788 allows maturation and duplication of pBBs after a lag of 2 h [23]. Mature basal bodies (mBBs) were stained with YL1/2 antibody (that recognizes TbRP2 protein in the transition zone) [17]. Both mBBs and pBBs were detected with anti-SAS6 antibody [15], since they both possess cartwheel protein SAS6 [36,37] (Fig 4A and 4B).

In cells with 1 mBB and 1 pBB (Fig 4C), the median distance between mBBs and pBBs was 421 nm (the 5^th^-to-95^th^ percentile range was 321-to-597 nm) in control (*i*.*e*., DMSO-treated) trypanosomes (Fig 4C, S2A Table), and 401 nm (with a 5^th^-to-95^th^ percentile range of 277-to-535 nm) for AEE788-treated cells (Fig 4C). Differences between the distribution of distances was statistically significant (p = 0.024, Mann-Whitney U test). We tracked changes in separation of basal bodies from 1.5–3 h after AEE788 was rinsed off, because S-phase entry begins 1 h after washing off the drug and probasal body maturation is detected between 2–3 h thereafter [23]. The median separation between mBBs and pBBs decreased to 377 nm at 1.5 h, and then increased to 443 nm at the 2-h point (Fig 4C, S2A Table). The difference in the median distances at 1.5 h and 2 h was statistically significant (p = 9.1*10^−12^, Mann-Whitney U test). Despite these statistically significant differences in medians, we are reluctant to make major biological inferences from the data, because of extensive overlap of distances in the 5^th^-to-95^th^ percentile (S2A Table).

We next determined distances between pro-basal bodies in cells with two mature basal bodies (Fig 4D, S2B Table). At the end of the AEE788 incubation, pBBs were separated by 895 nm (median) (5^th^-to-95^th^ percentile range = 388-to-1706 nm), whereas in DMSO-treated controls pBBs were separated by 905 nm (5^th^-to-95^th^ percentile range was 428-to-1425 nm); this difference was not significant statistically (p = 0.93, Mann-Whitney U test) compared to the AEE788-treated cells. After 1.5 h of recovery from AEE788 treatment, the median distance between two pro-basal bodies rose to 1122 nm, an increase that was statistically significant (p = 0.021, Mann-Whitney test) compared to the distance at 0 h. At two hours post-drug release, the median distance between pro-basal bodies was 1015 nm (5^th^-to-95^th^ percentile range = 312 nm-to-1524 nm). The difference in the distances between 1.5 and 2 h recovery time points was not statistically significant. At 3 h, the median pBB distance was 844 nm (5^th^-to-95^th^ percentile range 445-to-1169 nm). The decrease in distances between pro-basal bodies at 1.5 h and 3 h was statistically significant (p = 2.4*10^−5^; Mann-Whitney U test) (S2B Table).

Finally, we determined distances between pairs of mBBs associated with single (undivided) kDNAs (Fig 4D, numbers in red, S2C Table). Following AEE788 treatment, mBBs were separated by 1336 nm (5^th^-to-95^th^ percentile range 611–1922 nm). In trypanosomes incubated with DMSO, mBBs were separated by 1375 nm (median; 5^th^-to-95^th^ percentile range was 783-to-1949 nm). The difference in mBB separation between these two populations was not significant statistically (Mann-Whitney U test). After 1.5 h of release from AEE788 treatment, the median separation increased to 1535 nm (5^th^-to-95^th^ percentile range = 683–2306 nm), a nonsignificant change compared to data from trypanosomes at the end of exposure to AEE788 (S2C Table). Between 1.5 and 2 h of cell recovery from AEE788 exposure, the median separation of mBBs was 1511 nm (5^th^-to-95^th^ percentile range was 794–2033 nm). At 3 h post -AEE788 withdrawal, median separation between mBBs was 1304 nm (5^th^-to-95^th^ percentile range = 864–1700 nm), which was statistically significant when compared to distances measured at both 1.5 h recovery (p = 0.013, Mann-Whitney U test) and 2 h recovery (p = 0.0074, Mann-Whitney U test) (S2C Table).

In summary, we documented separation of pBBs from mBBs (Fig 4C), as well as their distance-dependent maturation (Fig 4D). The surprising results are; (i) nascent pBBs are found > 400 nm from mBBs (S2A Table), and (ii) maturation normally occurs after pBBs separate > 895 nm from mBBs (S2B Table). These data indicate that current models of basal body biogenesis in which pBBs mature in close proximity to mBBs may need to be revisited.

### Distances between mBBs is reduced after knockdown of TbCK1.2

Separation of basal bodies has been proposed as a mechanism for segregation (which encompasses division as well as partitioning of kDNA into daughter cells [10,30]) of kinetoplasts [1,13,20]. For this reason, we determined whether separation of mature basal bodies (mBBs) was compromised in 1K2N trypanosomes, by measuring inter-basal body distances using antibody YL1/2 [17]. This analysis was restricted to 1K2N cells with two mBBs.

**Fig 5:**
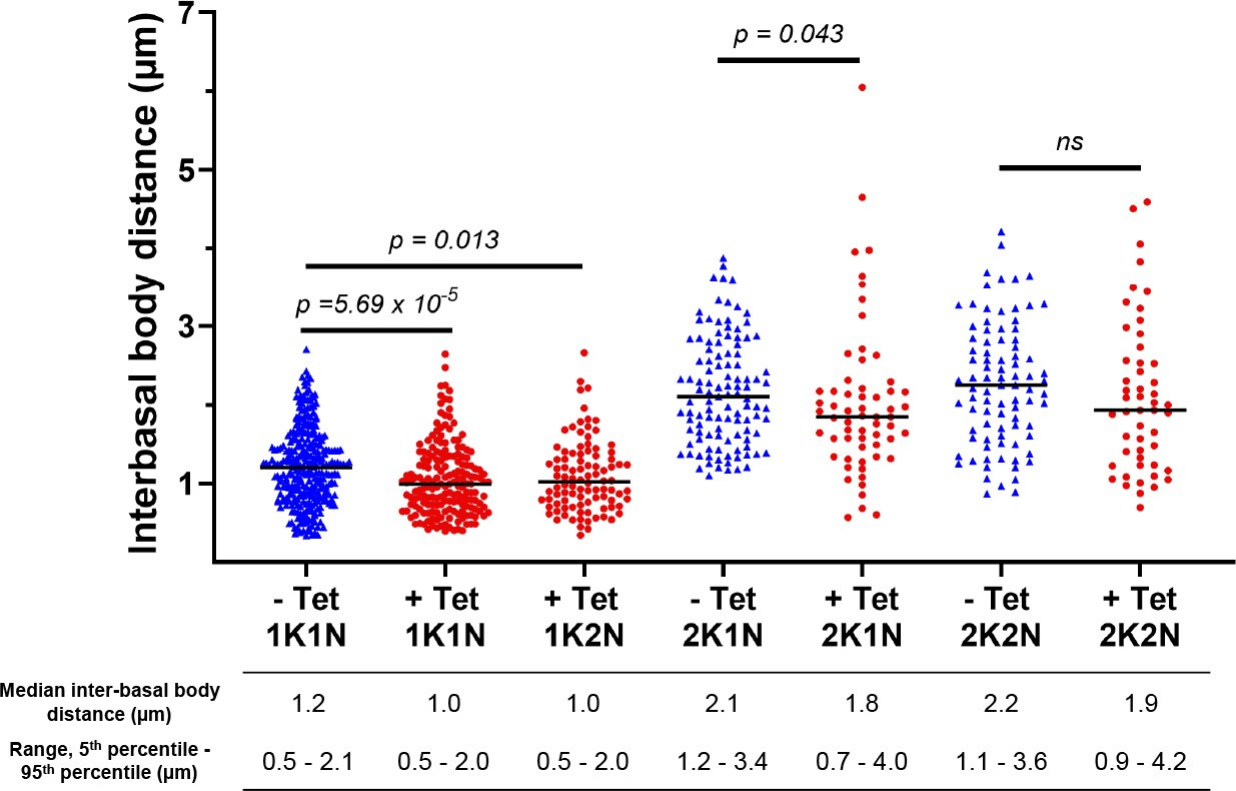
Basal body separation after knockdown of TbCK1.2. Knockdown of TbCK1.2 was induced with tetracycline (1 μg/mL for 24 h), and the distance between mature basal body pairs was measured. Antibody against TbRP2 (YL1/2) was used to identify basal bodies. ImageJ was used to measure inter-basal body distances (distance between two YL1/2^+^ mBBs) in TbCK1.2 RNAi cells cultured in the absence (-Tet) or presence of tetracycline (+Tet). A Mann-Whitney U test was used to determine whether differences in inter-basal body distances in control and TbCK1.2 RNAi cells were statistically significant; p values are noted on the graph. Data presented is an aggregate from six biological replicates (n = 302 (-Tet 1K1N), 183 (+Tet 1K1N), 96 (+Tet 1K2N), 111 (-Tet 2K1N), 61 (+Tet 2K1N), 88 (-Tet 2K2N), 51 (+Tet 2K2N)). Descriptive statistics are provided beneath the corresponding data on each graph.

In control 1K1N (-Tet) trypanosomes inter-basal body distances ranged from 0.3 μm to 2.7 μm with a median of 1.2 μm (Fig 5). That distance increased by 1 μm in both 2K1N (2.1 μm) and 2K2N (2.2 μm) trypanosomes (Fig 5). After TbCK1.2 knockdown, the median distance between basal bodies in 1K1N trypanosomes was 1.0 μm, instead of 1.2 μm (*p* = 5.7x10^-5^, Mann-Whitney U test) (Fig 5). In 1K2N trypanosomes the median inter-basal body distance (1.0 μm) was indistinguishable from that of knockdown 1K1N cells but was less than control 1K1N cells (*p* = 0.0133, Mann-Whitney U test) (Fig 5). Comparison of cells with one or two kinetoplasts following knockdown of TbCK1.2 showed that mBBs in trypanosomes with two kinetoplasts (2K1N) were separated by twice the distance found in trypanosomes with one kinetoplast (1K1N). The median inter-basal body distances were 1.8 μm (2K1N) and 1.9 μm (2K2N). By comparison those distances were 2.1 μm and 2.2 μm, respectively in control cells (Fig 5). The difference in separation of basal bodies between control and knockdown 2K1N trypanosomes was statistically significant (*p* = 0.0426, Mann-Whitney U test) but the difference between control and knockdown 2K2N cells was not (*p* = 0.0967, Mann-Whitney U test).

Overall, these data are consistent with successful separation of mBBs in *T*. *brucei* after knockdown of TbCK1.2, despite the decreased median inter-basal body distances, since the range of inter-basal body distances (5^th^-to-95^th^ percentile) are practically identical before and after knockdown of TbCK1.2. We conclude that separation of mBBs *per se* is not sufficient for division of kinetoplasts, since 1K2N cells fail at scission of kinetoplasts although they contain clearly-separated mBBs. Nevertheless, the distance between separated mBBs decreased by 0.2 μm (median) after knockdown of TbCK1.2, suggesting that mBBs may need to separate beyond 1.2 μm before division of kDNA takes place in a trypanosome. Two hypotheses are proposed to reconcile these data (see Discussion).

Our data also show that, unlike basal bodies in insect stage (procyclic) *T*. *brucei* [14,29], mBBs in bloodstream trypanosomes do not migrate further apart in 2K2N (compared to 2K1N) cells (Fig 5).

Basal bodies nucleate axonemal microtubules of flagella/cilia [20]. For that reason, we evaluated competence of basal bodies in 1K2N trypanosomes to form flagella (S4A Fig). The majority (75%) of 1K2N cells had two flagella (S4B Fig) indicating that the basal bodies retain competence for microtubule nucleation. A small percentage of trypanosomes had more than two flagella, indicating that some supernumerary basal bodies (Fig 3) produce flagella.

### TbCK1.2 is detected in the cytoplasm

**Fig 6:**
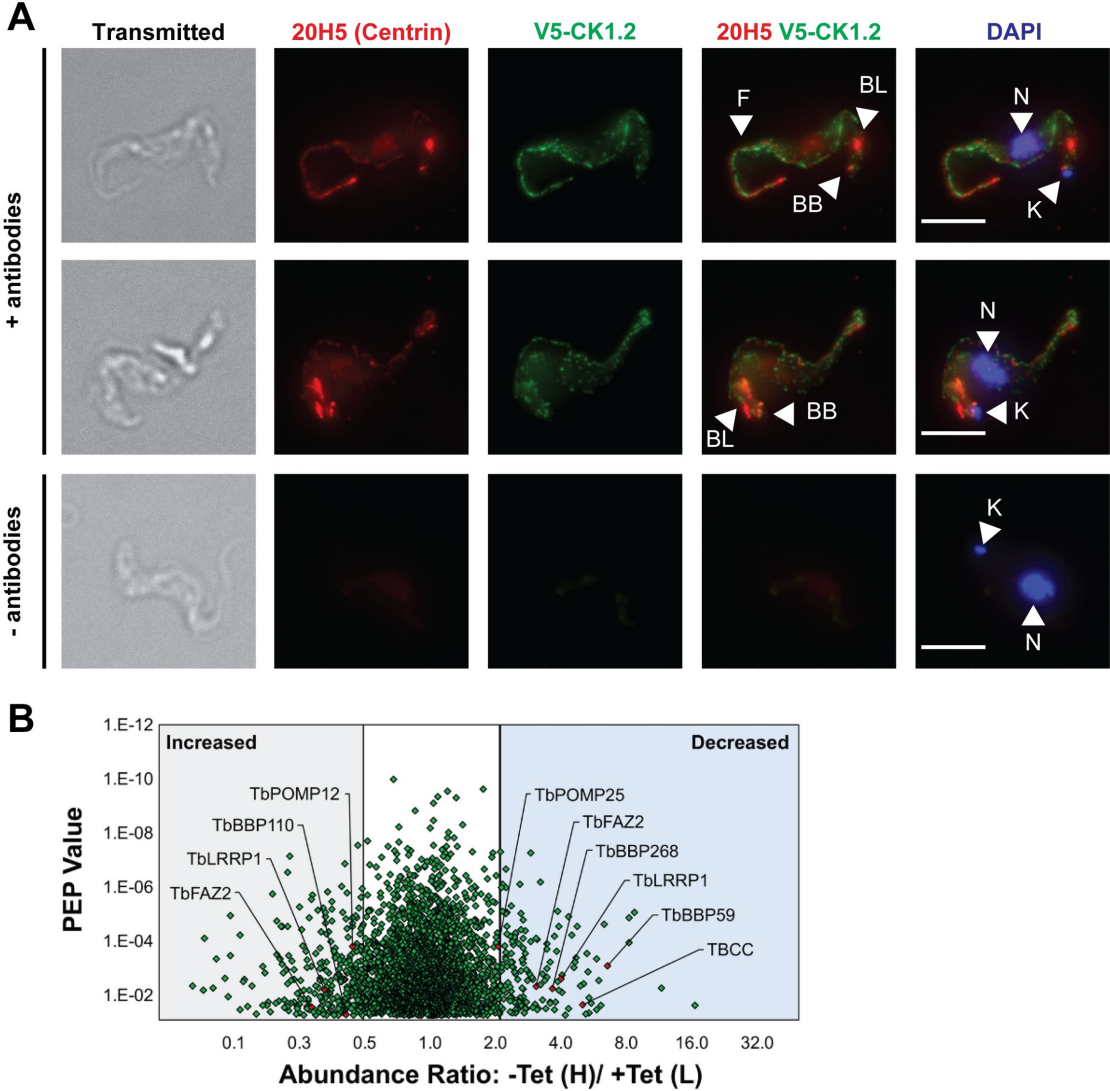
Intracellular location of TbCK1.2: Identification of candidate TbCK1.2-pathway proteins. **(*A***) Trypanosomes expressing TbCK1.2 tagged at its N-terminus with V5 epitope were fixed with methanol and probed with anti-V5 antibody. Anti-centrin antibody 20H5 was used to stain basal bodies and bilobes, and DAPI was used to stain DNA. Control images show a V5-TbCK1.2 tagged line without addition of primary antibodies. Arrowheads indicate flagellum (F), basal bodies (BB), bilobes (BL), nuclei, (N), and kinetoplasts (K). Scale bar = 5 μm. (***B***) Following knockdown (24 h) of TbCK1.2, SILAC phosphoproteomics was used to identify candidate TbCK1.2-pathway proteins. The abundance ratio (H/L) of identified phosphopeptides is plotted as a function of their posterior error probability (PEP) value. Only peptides with a PEP score of 5 x 10^−2^ (5% chance of error), or lower, are presented. Grey area represents phosphopeptides with a 2-fold or greater increase in abundance. Blue shading indicates phosphopeptides that decreased in abundance 2-fold or greater.

We considered a possibility that TbCK1.2’s effect on kinetoplast division could be explained, at least in part, by its intracellular location. Using a V5-epitope tagged TbCK1.2 RNAi line (S2A Fig) we localized TbCK1.2 to cytoplasmic puncta (S2E Fig and Fig 6). TbCK1.2 protein sequence lacks a mitochondrial targeting signal at its N-terminus that could have been disrupted by a V5-tag. In control experiments, similar data were obtained when a C-terminal HA-tagged version of TbCK1.2 was used in immunofluorescence studies (S2F Fig).

Since TbCK1.2 was not detected predominantly in mitochondria (Fig 6A), these data suggest that the effect of the enzyme on kDNA scission is most likely transmitted by other factors (*i*.*e*., effectors) (see next section).

### TbCK1.2-pathway proteins

To identify effector proteins in TbCK1.2-signaling pathways, we sought polypeptides whose phosphorylation changed after knockdown of TbCK1.2. We performed three independent phospho-proteomics studies, two label-free shotgun experiments [38,39] and one SILAC “target list” study [40,41].

Following knockdown of TbCK1.2 the abundance of 65 phospho-peptides (corresponding to 53 unique gene IDs [42]) decreased at least two-fold in each phospho-proteomic study, and 144 phospho-peptides (corresponding to 109 unique gene IDs) increased at least two-fold in each phospho-proteomic study, as compared to the uninduced controls (Fig 6B, S3 and S4 Tables). Phospho-peptides that changed in abundance after knockdown are considered “TbCK1.2-pathway proteins”; those that decreased in abundance are potential substrates of the enzyme.

### Some TbCK1.2-pathway proteins might localize to the mitochondrion

TbCK1.2 regulates division of kinetoplasts, which are inside mitochondria (Fig 1). However, the enzyme is found predominantly in the cytoplasm (Fig 6A). Since the vast majority of mitochondrial proteins are produced in the cytoplasm before their import into the organelle [43,44], we hypothesized that TbCK1.2’s modulation of kinetoplast division might involve “effector proteins” that are phosphorylated in the cytoplasm prior to their movement into the mitochondrion. This hypothesis has precedence: Three cytoplasmic protein kinases in *Saccharomyces cerevisiae* have substrates that are imported into mitochondria [45–47]. Consequently, we inquired whether any TbCK1.2-pathway proteins were potentially mitochondrial.

“TbCK1.2-Pathway Proteins” (i.e., 65 de-phosphorylated peptides (S3 Table) (corresponding to 53 gene IDs) and 144 hyper-phosphorylated peptides (S4 Table) (corresponding to 109 gene IDs) were combined into one data set, and were analyzed for possible mitochondrial association as follows. Gene identities (IDs) were compared to proteins that localize to mitochondria in trypanosomes (as reported by TrypTag, an *in vivo* protein-tagging and localization database [48]). Two *bona fide* mitochondrial proteins were found among TbCK1.2-Pathway proteins (S5A Table). In a second approach, TbCK1.2 pathway proteins were compared to two mitochondrial proteomes containing 1730 proteins [49,50] in TryTripDB (release 49 beta) [51]. Polypeptides found in both data sets were filtered by eliminating glycosomal or nuclear proteins [52,53], resulting in 13 proteins (S5B Table). (Proteins are imported post-translationally into nuclei and glycosomes [54,55]). Thus, there is a total of 15 mitochondrial proteins, two of which have been verified at the cellular level, among TbCK1.2-pathway proteins. Future work will address possible contributions of these proteins to kDNA division.

## Discussion

### Casein kinase TbCK1.2 has multiple functions in the African trypanosome

Protein kinases are considered “biological switches” that regulate physiological pathways instead of metabolic enzymes optimized to act on single substrates (reviewed in [56]). Typically, a single protein kinase affects multiple pathways in a cell. For example, JAK kinase is involved in IL2 synthesis [57], thyrotropin signaling [58], and centrosomal protein phosphorylation [59]. Similarly, vertebrate casein kinase 1δ stabilizes mature axons, [60], governs periodicity of mammalian circadian rhythms [61], and regulates cognitive-affective behavior in mice [62]. Protein kinases exhibit this myriad of functions because they have multiple substrates, some of which are effectors for pathways regulated by the enzymes [56]. Efforts to understand the contributions of protein kinases to biology have been most fruitful when single pathways are studied in detail to identify participants that are eventually ordered into signaling cascades, as shown for [57,58,63], EGFR [64–66], and CDKs [67–69].

In *T*. *brucei*, we find that TbCK1.2 affects cytokinesis (Fig 1), separation of mBBs (Fig 5), and scission of kDNA (Fig 2). Following the lead of investigators in other biological systems [57,58,63,70–72] (discussed above), we focus this manuscript on a single pathway affected by TbCK1.2, kinetoplast division (Figs 1 and 2). This decision is not meant to minimize the importance of other pathways affected by TbCK1.2. Neither is it a suggestion that failure of kinetoplast scission after knockdown of TbCK1.2 causes the other effects mentioned above. Instead, the decision is an acknowledgement of the futility of attempting to provide a comprehensive account of all three physiological pathways affected by the enzyme in one publication. Our working hypothesis is that all pathways affected by TbCK1.2 are impacted concurrently, because the enzyme is found in multiple regions of the cell (Figs 6, S2E and S2F) where it engages different effector proteins (S3 Table) for each of the pathways affected by the enzyme.

### Kinetoplast division factor hypothesis

A kinetoplast biogenesis cycle has five steps, minimally; kDNA synthesis, selection of scission sites, cleavage/scission, separation of kinetoplasts, and sorting of cleaved kDNAs (S1 Fig) (see Introduction for definition of terms). Division (*i*.*e*., scission/cleavage and initial separation) of kDNA is poorly characterized; no protein that mediates the process has been identified to date (reviewed in [12]).

In this report we show that mutant 1K2N trypanosomes obtained after knockdown of TbCK1.2 (Fig 1) have two well-separated basal bodies (Figs 3 and 5 and S5) and yet fail to divide kDNA (Fig 1). Hence knockdown of TbCK1.2 de-couples basal body separation from division of kinetoplasts, so that basal bodies move apart without scission of kDNA. Separation of basal bodies is not sufficient to divide a kinetoplast, although that event precedes segregation of kinetoplasts [1,13,14,20]. (The original use of the word “segregation” in kinetoplastid biology [10] referred to the process that we term “scission” (S1 Fig) [11]. However, “segregation” is now used for all events associated with kDNA inheritance [10–12].

Based on our data (summarized above) we propose that trypanosomes with one kinetoplast and one nucleus can have a post-basal body separation “kDNA intermediate” containing uncleaved kDNA (K_U_). The intermediate (K_U_) is short-lived under normal circumstances. Conversion of K_U_ into two cleaved kDNA networks (in 2K1N trypanosomes) is arrested after knockdown of TbCK1.2, making it possible to detect the normally elusive 1K_U_1N intermediate (Fig 1E and 1F). In DAPI staining of kDNA, the intermediate may be detected as a 1K1N trypanosome with “over-replicated” kDNA (Fig 1E); quantitative electron microscopy studies document scission failure of K_U_ (Fig 2A). With time, a 1K_U_1N produces 1K_U_2N trypanosomes after mitosis, (Fig 1D and 1F).

**Fig 7:**
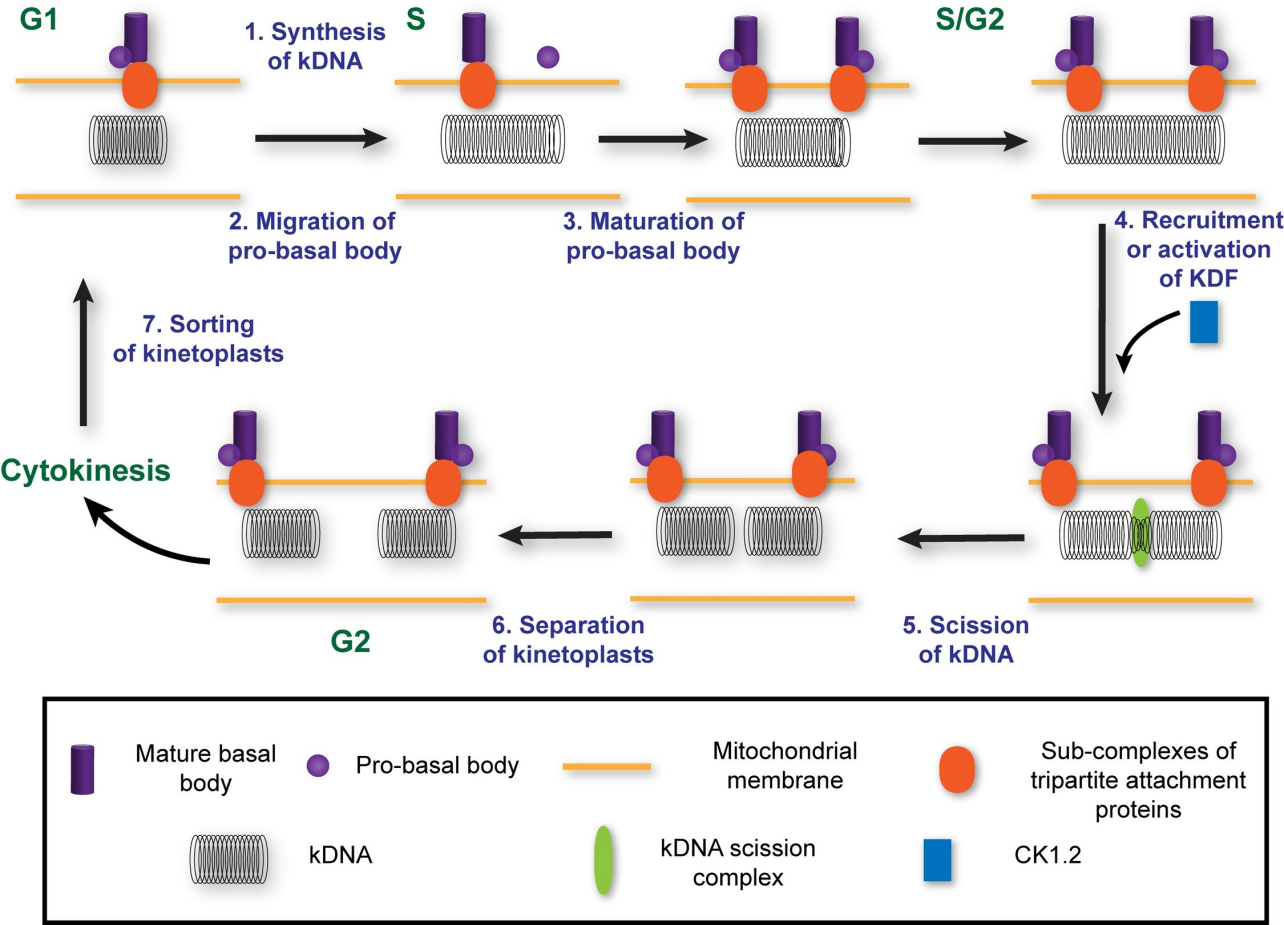
Kinetoplast division factor hypothesis. In G1, trypanosomes have one basal body and a single kinetoplast. During S-phase, kDNA synthesis occurs and the probasal body migrates away from the mature basal body (Step 1 and 2). After separating by a distance >895 nm, the probasal body completes maturation (step 3) producing 1K1N trypanosomes with 2 mature basal bodies that are found near opposite ends of a kinetoplast containing uncleaved double-length kDNA [23]. We propose that presence of two basal bodies each at a pole of kinetoplast “licences” kDNA division. Subsequently, kinetoplast division factors (KDFs) are recruited close to, or into, the mitochondrion (Step 4). KDFs may recruit or activate a “kDNA cleavage/scission complex” to divide the kinetoplast (Step 5). In G2, separation of kinetoplasts is visible (Step 6), providing microscopic evidence of kDNA scission. Sorting of kinetoplasts into daughter trypanosomes occurs at cytokinesis (Step 7). Depiction of a “kDNA cleavage/scission complex” is hypothetical. Knockdown of TbCK1.2, or other KDFs, prevents scission of kDNA (see Fig 2). Molecular functions of other KDFs remain to be discovered. TAC-protein sub-complexes are projected to might mediate scission site selection and/or sorting of kinetoplasts (reviewed in [11]).

We incorporate these concepts (above) into a kinetoplast division factor (KDF) hypothesis (Fig 7). Previous hypotheses addressed “segregation” of kinetoplasts [1,10,12,30]. The term “segregation” is applied to multiple stages of kinetoplast inheritance; it is not equivalent to “division”, which is a specific step in biogenesis of kinetoplasts (Fig 7). Data justifying our hypothesis include the following; (i) separation of mature basal bodies is not sufficient to divide kinetoplasts (Figs 2 and 5); and (ii) kDNA synthesis was completed in 1K2N trypanosomes (Fig 1E). Lastly, we highlight candidate KDFs, twelve proteins whose knockdown yields 1K2N cells, namely KMP-11 [73], TbCEP57 [15], GCP2 (γ-tubulin) [74], PK53 [24], CRK9 [24], ULK [24], RDK2 [24], PF16 [75], HslVU [76], AP1-γ subunit [11], UMSBP1, and UMSBP2 [77]. These facts are accommodated in a KDF hypothesis (below).

In G1, trypanosomes have one mature basal body and one pro-basal body (Fig 7). During S-phase, kDNA synthesis (Step 1) is accompanied by separation of the pro-basal body from the mature basal body (Step 2), and maturation of pro-basal bodies (Step 3) [23,78] producing trypanosomes with two basal bodies and a double-length kDNA (Figs 1E and 2A). Mature basal bodies are separated between 0.55-to-2.11 microns (Fig 5). KDFs [11] are recruited (or activated) when mBBs are separated greater than 1.2 microns (Step 4), leading to scission of kDNA (Step 5). Basal body separation beyond a threshold of 1.2 microns may be a “licensing step” for scission of kDNA when KDFs are either activated or recruited to kinetoplasts. In G2, cleaved and separated kDNAs are visible microscopically (Step 6), and are sorted into daughter trypanosomes during cytokinesis (Step 7).

### Phenotypes accompanying knockdown of genes for KDFs or Tripartite Associated Complex (TAC)-associated proteins are distinguishable

Some properties of kinetoplasts in cells where TbCK1.2 (a KDF) was knocked down (Figs 1, 1E and 2) appeared to resemble those obtained after knockdown of TAC-associated proteins (TACAPs) [12]. A closer examination shows that mutants of KDFs and TAC-associated proteins have different properties. First, early phenotypes of kDNAs (*i*.*e*., observed within 24-h after knockdown of a gene in bloodstream *T*. *brucei*) are distinguishable between KDFs and TAC-associated proteins (discussed in [11]). KDF loss prevents scission of kDNA (Figs 1 and 2) whereas knockdown of TACAPs, best illustrated by RNAi of p166, the first reported TACAP [79], does not prevent cleavage of kDNA [11]. Second, we observed an increase in 2K2N (post-mitotic) trypanosomes after knockdown of TbCK1.2 (Fig 1, and see first paragraph of Discussion), pointing to defective cytokinesis, whereas knockdown of TACAPs does not inhibit cytokinesis [12]. Third, KDF knockdown reduces separation of mBBs whereas RNAi of TAC genes does not shorten distances between basal bodies. Finally, TAC gene mutations lead to loss of kDNA from proliferating trypanosomes whereas KDF knockdown is not associated with loss of kinetoplasts from *T*. *brucei*.

### Candidate effector proteins for TbCK1.2 regulation of kinetoplast scission

Although TbCK1.2 is a KDF (Fig 1), the protein is not detected selectively at the kinetoplast (Fig 6A). Therefore, TbCK1.2’s modulation of kinetoplast scission is likely to be mediated by “effector proteins” that localize to mitochondria.

In *T*. *brucei* we found fifteen putative mitochondrial proteins among TbCK1.2-pathway proteins (S5 Table). This observation is not unlike that in *Saccharomyces cerevisiae* where three cytoplasmic protein kinases have substrates that are imported into mitochondria [45–47], and a CK1 regulates activity of the protein import pore of mitochondria [80]. In future studies, will test whether or not the trypanosome proteins localize to mitochondria, and whether their knockdown (or overexpression) affects scission of kDNA.

### Experimental procedures

#### Parasite cultures

Bloodstream *T*. *brucei* CA427, single marker (SM) [81] RUMP528 [Leal et al 2001] or TbCK1.2 RNAi cell lines (see Supplemental Materials) were cultured as described [78].

#### Enumeration of kinetoplasts and nuclei after DAPI staining

Following genetic knockdown of TbCK1.2, cells (~1.5 x 10^6^) were stained with DAPI [78]. To induce knockdown, the TbCK1.2 RNAi line was incubated in the presence or absence of tetracycline (1 μg/ml) for 12, 18, or 24 hours. For the 12 and 18 h time points, three independent experiments were conducted (n = 116–160 trypanosomes/experimental sample). For the 24 h time point, four independent experiments were performed (125–150 trypanosomes analyzed/experiment).

#### Quantitation of fluorescence intensity from DAPI-stained kinetoplasts

After knockdown of TbCK1.2, images of DAPI-stained control and RNAi-treated trypanosomes were acquired using under the same conditions using a DeltaVision II microscope system. Additionally, the brightness and contrast settings of display images during post-processing were kept identical. Using ImageJ [82], a box was drawn over each kinetoplast and the sum of the pixels in the selection was measured (raw integrated density). To control for background fluorescence, a box with the same dimensions used for each kinetoplast was drawn at two areas near the organelle of interest, and the raw integrated density determined. The average of the two background fluorescence measurements was then subtracted from the integrated density of the respective kinetoplast. Cells were pooled from three independent experiments. For the 12 hour time point, 1K1N minus Tet n = 183, 1K1N plus Tet n = 198.

#### Transmission electron microscopy (TEM)

TEM of kinetoplast network was performed as described previously [83] with modifications. TbCK1.2 RNAi cells were induced for 24 h with tetracycline (1μg/ml). Trypanosomes (1x10^8^) were harvested from induced and uninduced cultures, and washed once with 15 ml of chilled PBS-G (glucose 10 mM). Cells were resuspended in 5 ml of fixative solution; 0.07 M cacodylate buffer (pH 7.4) containing 2% paraformaldehyde (EM grade) and 0.5% glutaraldehyde (EM grade), and incubated at 4°C for 1 h. Cells were washed twice with cacodylate buffer (0.07 M, pH 7.4), and were encapsulated in 100 μl of 4% low-melting-point agarose at 4°C for 2 h. The agarose enrobed trypanosomes were post-fixed with 4% aqueous OsO_4_ for 1 h, followed by dehydration in an ascending series of ethanol (25, 50, 75, 85, 95 and 100%, 15 mins at each step), and anhydrous acetone (10 mins, two times). After the dehydration, trypanosomes were incubated with an ascending series of Eponate 12 resin solution (Ted Pella Inc.) in acetone (25, 50, 75%) at room temperature for 2 h at each step. Finally, cells were embedded in Eponate 12 resin and polymerized at 60°C for 24 h. Ultrathin sections (60 to 70 nm) were cut, transferred onto copper grids, and stained with 2% aqueous uranyl acetate for 5 min. Sections were visualized with a JEOL JEM-1011 transmission electron microscope at 80 kV and 10,000 X magnification.

#### Quantitation of kinetoplast network length from TEM images

For both uninduced and induced trypanosomes (*i*.*e*., TbCK1.2 RNAi), 632 kinetoplast images were captured from three independent experiments (200 from first experiment, and 216 from second and third experiments) for quantitation. The length and width of each kinetoplast network was obtained using a measurement line tool in Fiji software [84].

#### Immunofluorescence assays

For detection of TbCK1.2, basal bodies, and flagella, trypanosomes (8 x 10^5^) were fixed with methanol or paraformaldehyde and labeled [78] with the appropriate antibodies (see Supplemental Material for antibody details). Double-staining with YL1/2 and anti-TbSAS6 was used to determine basal body number. Three biological replicates were performed (n = 108-128/sample). Anti-PFR2 was used to enumerate flagella number in three independent experiments (n = 96-130/sample). Anti-V5 antibody was used to detect V5-TbCK1.2. The YL1/2 antibody was used to detect basal bodies, and the 20H5 antibody to detect basal bodies and bilobes when localizing TbCK1.2. For visualization by fluorescence microscopy, mCLING was added to a final concentration of 1 μM, and cells were incubated on ice in the dark for 60 s and then fixed with 100 μL 4% PFA/0.05% glutaraldehyde and 0.01% saponin [6]. Twenty μL of the solution was transferred to a coverslip coated with poly-L-lysine, and dried on a bead bath heated to 50 ^o^C. The cover slips were immersed in PBS for 5 min for re-hydration, briefly rinsed with deionized H_2_O, gently dried with a Kim-Wipe, and mounted on a slide with VectaShield mounting medium (Vector Laboratories, Burlingame, CA) containing 5 μM 4’,6-diamidino-2-phenylindole (DAPI). Images were captured on an EVOS-FL microscope (ThermoFisher), and numbers of kinetoplasts and nuclei per cell (n = 150 per experiment) were counted. Three independent experiments were performed. Statistical analysis was performed as described below.

#### Measurement of inter-basal body distances

The distance between basal bodies in trypanosomes with two mature basal bodies was determined by drawing a line between the center of YL1/2-positive basal bodies and measuring the distance using ImageJ. Trypanosomes from five independent experiments were analyzed (97 uninduced 1K1N cells, 94 induced 1K1N cells, 95 induced 1K2N cells, 81 uninduced 2K1N cells, 61 induced 2K1N cells, 84 uninduced 2K2N cells, 51 induced 2K2N cells).

The distance between pro-basal body and mature basal body in trypanosomes with one mature basal body, and distance between two pro-basal bodies in cells that had two mature basal bodies was determined by drawing a line between centers of anti-SAS6-positive objects, and measuring the distance using ImageJ. All cells analyzed had one kinetoplast.

For AEE788 experiments, the numbers of cells with one mature basal body were as follows: 41 from DMSO treated group, 131 from AEE788 treated group, 99 cells from population treated with AEE788 and allowed to recover from drug pressure for 1.5 h, 106 cells from 2 h recovery, and 62 cells from 3 h recovery. For cells with two mature basal bodies, numbers analyzed were respectively: 58 cells from DMSO treatment, 29 from AEE788 treatment, 16 from 1.5 h recovery, 32 from 2 h recovery, and 92 from 3 h recovery group.

#### Western blotting

Total cell lysate from trypanosomes (8 x 10^5^ per sample) was used for western blotting [85]. A Stain-Free labeled gel was activated [86,87] before transfer of proteins to a PVDF membrane for normalization of total protein (see Supplemental Material).

#### SILAC and label-free preparation of trypanosome peptides for LC-MS/MS

Three mass spectrometry experiments were performed. Label-free phosphopeptides were isolated and analyzed in two biological replicates as described in *Supplemental Materials*. An inclusion list [88] was used during analysis of the second label-free experiment (see Supplemental Material). Additionally, a tetracycline-inducible TbCK1.2 RNAi line was cultured in HMI-9 medium modified for SILAC [89,90]. Induced (light medium) and uninduced (heavy medium) trypanosomes (3 x 10^7^ cells per sample) were combined and processed as described [91]. Phospho-peptide enrichment and LC-MS/MS analysis is described in *Supplemental Material*. Spectral counts from the two label-free experiments were combined, and phospho-peptides that showed at least a 2-fold decrease (or increase) in SILAC and the label-free strategies were considered putative TbCK1.2-pathway proteins.

### Statistical analysis

Unless otherwise stated, Excel (Microsoft) and Graphpad Prism were used for Student’s t test. Chi-squared (x^2^), Mann-Whitney, and one-way ANOVA tests were executed using GraphPad Prism. For all statistical analysis, α = 0.05. Exact p-values for most statistical tests are calculated in Prism to 15 significant digits. In some cases, exact p-values were unavailable due to being smaller than 1x10^-15^. Exact p-values for Dunnett’s multiple comparisons test are calculated to 4 significant digits. Exact p-values smaller than 1x10^-3^ are not calculated.

## Supporting information

S1 Fig The kinetoplast duplication cycle. Basal bodies and subcomplexes of tripartite attachment complex (TAC) proteins are found near kinetoplasts. In G1, trypanosomes have one basal body and a single kinetoplast. During S-phase, kDNA synthesis occurs (Step 1), and a second mature basal body is produced via maturation of the probasal body (Step 2). Both mature basal bodies gain a new probasal body companion. Division of the kDNA network (Step 3) takes place before separation of the kinetoplasts is visible (Step 4). During cytokinesis kinetoplasts are sorted into daughter trypanosomes (Step 5). Sub-complexes of TAC proteins [1] might mediate kDNA scission site selection and/or sorting of kinetoplasts (reviewed in [2]).
(TIF)

S2 Fig Reduction in protein level of TbCK1.2 by RNA interference. One allele of TbCK1.2 was tagged with a V5 epitope (N-terminal) in a cell line harboring a tetracycline-inducible TbCK1.2 RNAi construct. Trypanosomes were incubated in the absence (-Tet) or presence (+Tet) of tetracycline (1 μg/ml) for 24 h. (*A*) Western blot using an anti-V5 antibody to probe lysate from uninduced (-Tet) and induced trypanosomes (+Tet). Total protein was monitored with a Stain-Free protocol [3, 4]. (*B*) Plot presents the average normalized band intensity (see *Materials and Methods*) of V5-TbCK1.2 (39 kDA) in three biological replicates. A Student’s t-test was used to test the statistical significance of the difference in relative V5 band intensity in uninduced and induced TbCK1.2 RNAi cell populations. (*C*) Trypanosome density was determined 12 h, 16 h, or 24 h after the addition of tetracycline, starting from 5 x 10^4^ cells/ml. Average cell density and standard deviation of three independent biological experiments are shown. (*D*) V5-TbCK1.2 RNAi cell line was incubated in the absence (-Tet) or presence (+Tet) of tetracycline for 24 h. Cells were fixed in paraformaldehyde, and tagged V5-TbCK1.2 was detected with anti-V5 antibody. Scatter/violin plot indicates fluorescence intensity in whole cells with or without tetracycline. Intensity of V5-TbCK1.2 signal in cells was measured in three biological replicates using Fiji (33–58 cells per group in individual replicates). Distributions of fluorescence values were compared via a Mann-Whitney test. Bars indicate median and inter-quartile range. Dotted line represents mean V5-TbCK12 fluorescence in -Tet group. (*E*) Representative images from immunofluorescence assays performed with anti-V5 antibody. TbRP2, a basal body transition zone protein, was visualized using YL1/2 antibody. Bottom row shows tagged cells not exposed to primary antibodies. Scale bar = 5 μm. (*F*) One allele of TbCK1.2 was tagged with an HA epitope (C-terminal), and used for immunofluorescence assays with anti-HA antibody following paraformaldehyde fixation. TbRP2 protein was visualized using YL1/2 antibody. Scale bar = 5 μm.
(TIF)

S3 Fig Kinetoplast division monitored by electron microscopy. (*A*) Transmission electron microscopy images showing kinetoplast network duplication cycle in *T*. *brucei*. Panel *a* depicts a kinetoplast (K) of median length (~ 400 nm). Panel *b* is a representative image for elongated kinetoplast (Ke), whereas *c* and *d* show kinetoplasts at the scission and separation steps of division, respectively. Panel *e* shows two cleaved and separated kinetoplasts (K-1 and K-2), each surrounded by mitochondrial membranes (MtM). (*B*) *Widths of kinetoplasts are not affected by knockdown of TbCK1*.*2*. (a) A scatter plot showing the width of kinetoplast network in uninduced and induced population (24 h) of TbCK1.2 RNAi cell line. TEM images of kinetoplast were used for measurement of width, n = 100 for uninduced and induced population, ns; non-significant, Mann Whitney U test was used for statistical analysis. (b) Representative TEM images of kinetoplasts from uninduced and induced population of TbCK1.2 RNAi cell line. K; kinetoplast, MtM; mitochondrial membrane. Scale bar; 200 nm.
(TIF)

S4 Fig Flagella are formed by basal bodies in 1K2N trypanosomes. Knockdown of TbCK1.2 was induced with tetracycline. Cells were fixed with methanol and an antibody against TbRP2 (YL1/2) used to identify mature basal bodies. Flagella were detected with anti-PFR2 (against a paraflagellar rod protein). Kinetoplasts and nuclei were stained with DAPI. For all images the scale bar is 6 μm. (***A***) Representative staining of the TbRP2 (mature basal bodies) and PFR2 (flagellum) in TbCK1.2 RNAi cells cultured in the presence (+Tet) or absence of tetracycline (-Tet). Arrows indicate flagella, arrowheads indicate mature basal bodies. (***B***) The average percentage of 1K2N cells with the indicated number of flagella is shown, from three independent experiments (n = 96-130/experiment).
(TIF)

S5 Fig Basal body, flagella, and kDNA observed with superresolution microscopy in a 1K2N trypanosome. Membranes were stained with mCLING [5], basal bodies were labeled with YL1/2 antibody, and DNA was stained with DAPI. Maximum intensity projections of z-stack of images were acquired with an SR-SIM microscope.
(TIF)

S1 Table Descriptive statistics for kDNA intensity measurements before and after knockdown of TbCK1.2. Experimental details are presented in the legend to Fig 1B.
(DOCX)

S2 Table Inter-basal body distances following cell cycle synchronization. RUMP528 cells were treated with 5 μM of AEE788 or 0.1% (v/v) DMSO. After this, cells were released from drug treatment. Median distances and 95% confidence intervals of median between mature basal body and probasal body (for cells with a single mature basal body), and between two probasal bodies (for cells in which development of a second mature basal body has occurred) are displayed.
(DOCX)

S3 Table TbCK1.2 pathway proteins with decreased phospho-peptide abundance after knockdown of TbCK1.2. Following a 24-h knockdown of TbCK1.2, phospho-peptides were harvested from uninduced and induced cells and phospho-peptides enriched over an IMAC column (see materials and methods). Phospho-peptide abundance was calculated in each sample using a labeled proteomics (SILAC) (n = 1) and label-free approach (spectral counting (SC)) (n = 2). Phospho-peptides identified with decreased abundance (at least 2-fold) in each phosphoproteomics strategy are listed. Phosphorylation sites are indicated in red (PhosphoRS [6] value >79%). * indicates the number of phospho-sites which could not be accurately assigned. The fold change in phospho-peptide abundance, as compared to the uninduced control, is shown. ~99 indicates that the phospho-peptide was only present in the control or induced population, preventing calculation of an abundance ratio. All listed peptides had a PEP value (probability that spectra-peptide match was incorrect) of 6% or less. N/A indicates that the exact phospho-isoform of the indicated peptide was not identified. A control experiment comparing the abundance ratio of phospho-peptides from uninduced cells grown in heavy or light SILAC medium was performed. Peptides that showed a 2-fold change in abundance in both the control and experimental group are not reported as TbCK1.2 pathway proteins.
(DOCX)

S4 Table Putative TbCK1.2 effectors with increased phospho-peptide abundance after knockdown of TbCK1.2. Following a 24-h knockdown of TbCK1.2, phospho-peptides were harvested from uninduced and induced cells and phospho-peptides enriched over an IMAC column (see materials and methods). Phospho-peptide abundance was calculated in each sample using a labeled proteomics (SILAC) (n = 1) and label-free approach (spectral counting (SC)) (n = 2). Phospho-peptides identified with increased abundance (at least 2-fold) in each phosphoproteomics strategy are listed. Phosphorylation sites are indicated in red (PhosphoRS [6] value >79%). * indicates the number of phospho-sites which could not be accurately assigned. The fold change in phospho-peptide abundance, as compared to the uninduced control, is shown. ~99 indicates that the phospho-peptide was only present in the control or induced population, preventing calculation of an abundance ratio. All listed peptides had a PEP value (probability that spectra-peptide match was incorrect) of 6% or less. N/A indicates that the exact phospho-isoform of the indicated peptide was not identified. A control experiment comparing the abundance ratio of phospho-peptides from uninduced cells grown in heavy or light SILAC medium was performed. Peptides that showed a 2-fold change in abundance in both the control and experimental group are not reported as putative TbCK1.2 effectors.
(DOCX)

S5 Table Putative mitochondrial TbCK1.2 pathway proteins. “TbCK1.2-Pathway Proteins” (S4 and S5 Tables) were re-analyzed in search for mitochondrial proteins as follows. (A) Gene IDs for TbCK1.2-pathway proteins were compared to proteins that localize to the mitochondrion in the TrypTag database. (B) Gene IDs for TbCK1.2-pathway proteins were compared to two mitochondrial proteomes (combined) containing 1730 proteins [7, 8] available in TryTripDB (release 41) [9]. Polypeptides found in both data sets were filtered by eliminating proteins in glycosome or nucleus proteomes [10, 11], resulting in 21 proteins. (Proteins are imported post-translationally into both nuclei and glycosomes [12, 13]).
(DOCX)

S1 File Supplemental experimental procedures. (DOCX)
