# Supplementary figures and images for: Casein kinase TbCK1.2 regulates division of kinetoplast DNA, and movement of basal bodies in the African trypanosome
Source: PLoS One. 2021 Apr 16;16(4):e0249908. doi: 10.1371/journal.pone.0249908 (PMC8051774; doi:10.1371/journal.pone.0249908)

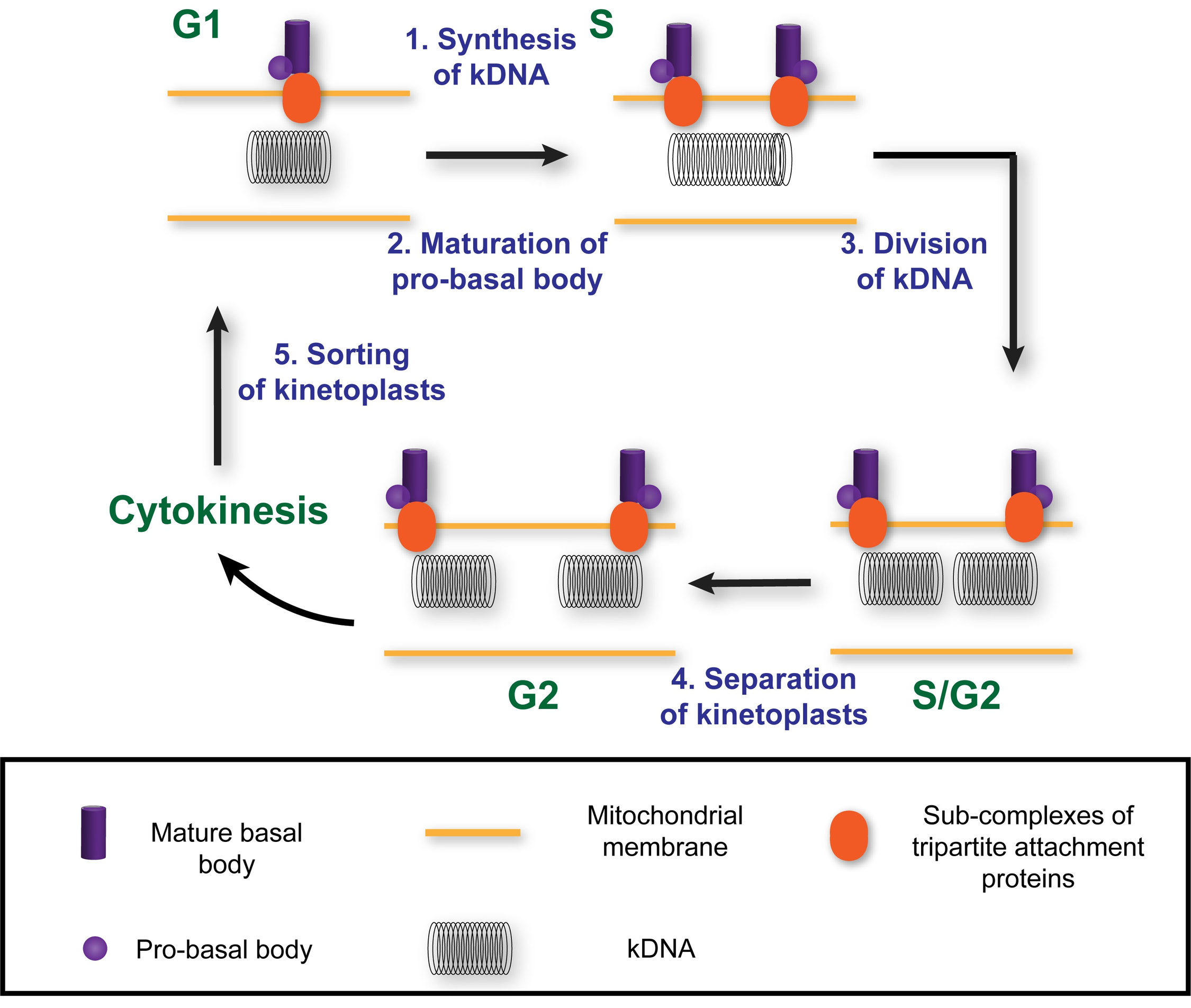

Supplement: S1 Fig — Basal bodies and subcomplexes of tripartite attachment complex (TAC) proteins are found near kinetoplasts. In G1, trypanosomes have one basal body and a single kinetoplast. During S-phase, kDNA synthesis occurs (Step 1), and a second mature basal body is produced via maturation of the probasal body (Step 2). Both mature basal bodies gain a new probasal body companion. Division of the kDNA network (Step 3) takes place before separation of the kinetoplasts is visible (Step 4). During cytokinesis kinetoplasts are sorted into daughter trypanosomes (Step 5). Sub-complexes of TAC proteins [1] might mediate kDNA scission site selection and/or sorting of kinetoplasts (reviewed in [2]). (TIF) [file pone.0249908.s001.tif]

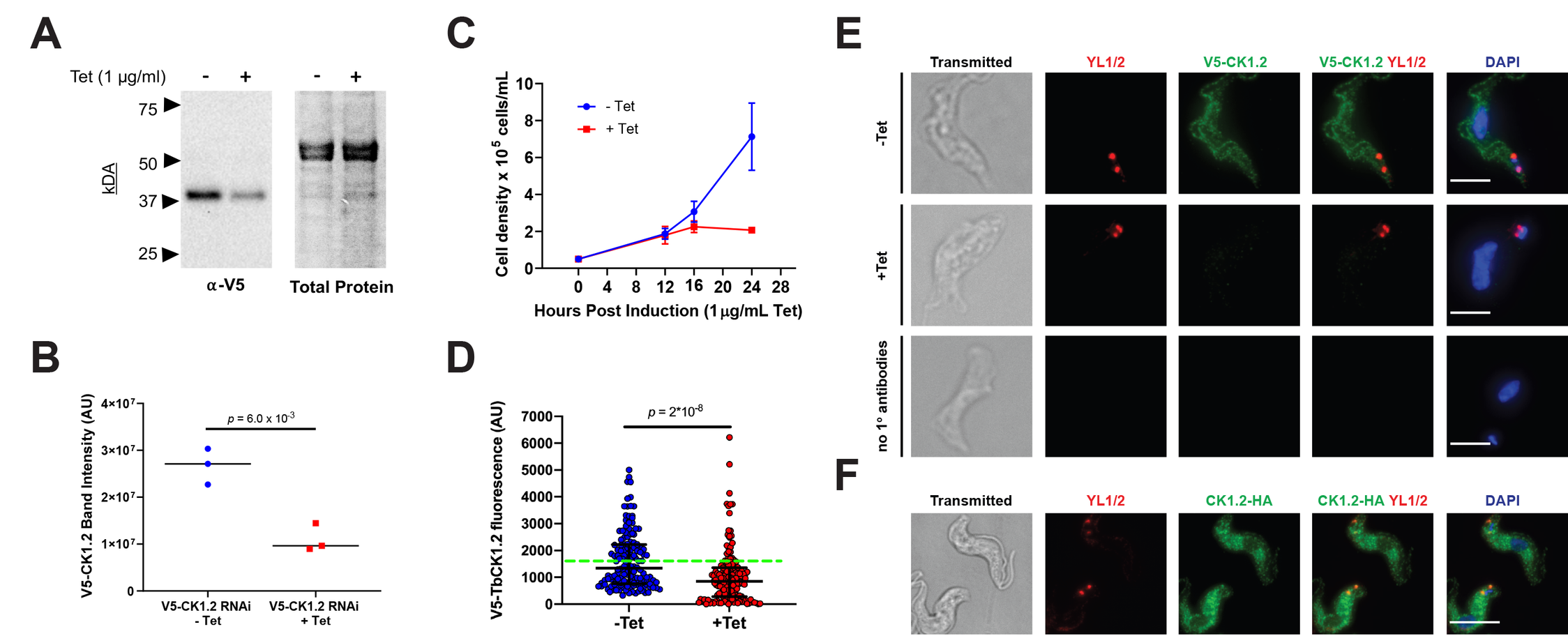

Supplement: S2 Fig — One allele of TbCK1.2 was tagged with a V5 epitope (N-terminal) in a cell line harboring a tetracycline-inducible TbCK1.2 RNAi construct. Trypanosomes were incubated in the absence (-Tet) or presence (+Tet) of tetracycline (1 μg/ml) for 24 h. (A) Western blot using an anti-V5 antibody to probe lysate from uninduced (-Tet) and induced trypanosomes (+Tet). Total protein was monitored with a Stain-Free protocol [3, 4]. (B) Plot presents the average normalized band intensity (see Materials and Methods) of V5-TbCK1.2 (39 kDA) in three biological replicates. A Student’s t-test was used to test the statistical significance of the difference in relative V5 band intensity in uninduced and induced TbCK1.2 RNAi cell populations. (C) Trypanosome density was determined 12 h, 16 h, or 24 h after the addition of tetracycline, starting from 5 x 10^4^ cells/ml. Average cell density and standard deviation of three independent biological experiments are shown. (D) V5-TbCK1.2 RNAi cell line was incubated in the absence (-Tet) or presence (+Tet) of tetracycline for 24 h. Cells were fixed in paraformaldehyde, and tagged V5-TbCK1.2 was detected with anti-V5 antibody. Scatter/violin plot indicates fluorescence intensity in whole cells with or without tetracycline. Intensity of V5-TbCK1.2 signal in cells was measured in three biological replicates using Fiji (33–58 cells per group in individual replicates). Distributions of fluorescence values were compared via a Mann-Whitney test. Bars indicate median and inter-quartile range. Dotted line represents mean V5-TbCK12 fluorescence in -Tet group. (E) Representative images from immunofluorescence assays performed with anti-V5 antibody. TbRP2, a basal body transition zone protein, was visualized using YL1/2 antibody. Bottom row shows tagged cells not exposed to primary antibodies. Scale bar = 5 μm. (F) One allele of TbCK1.2 was tagged with an HA epitope (C-terminal), and used for immunofluorescence assays with anti-HA antibody following paraformald [file pone.0249908.s002.tif]

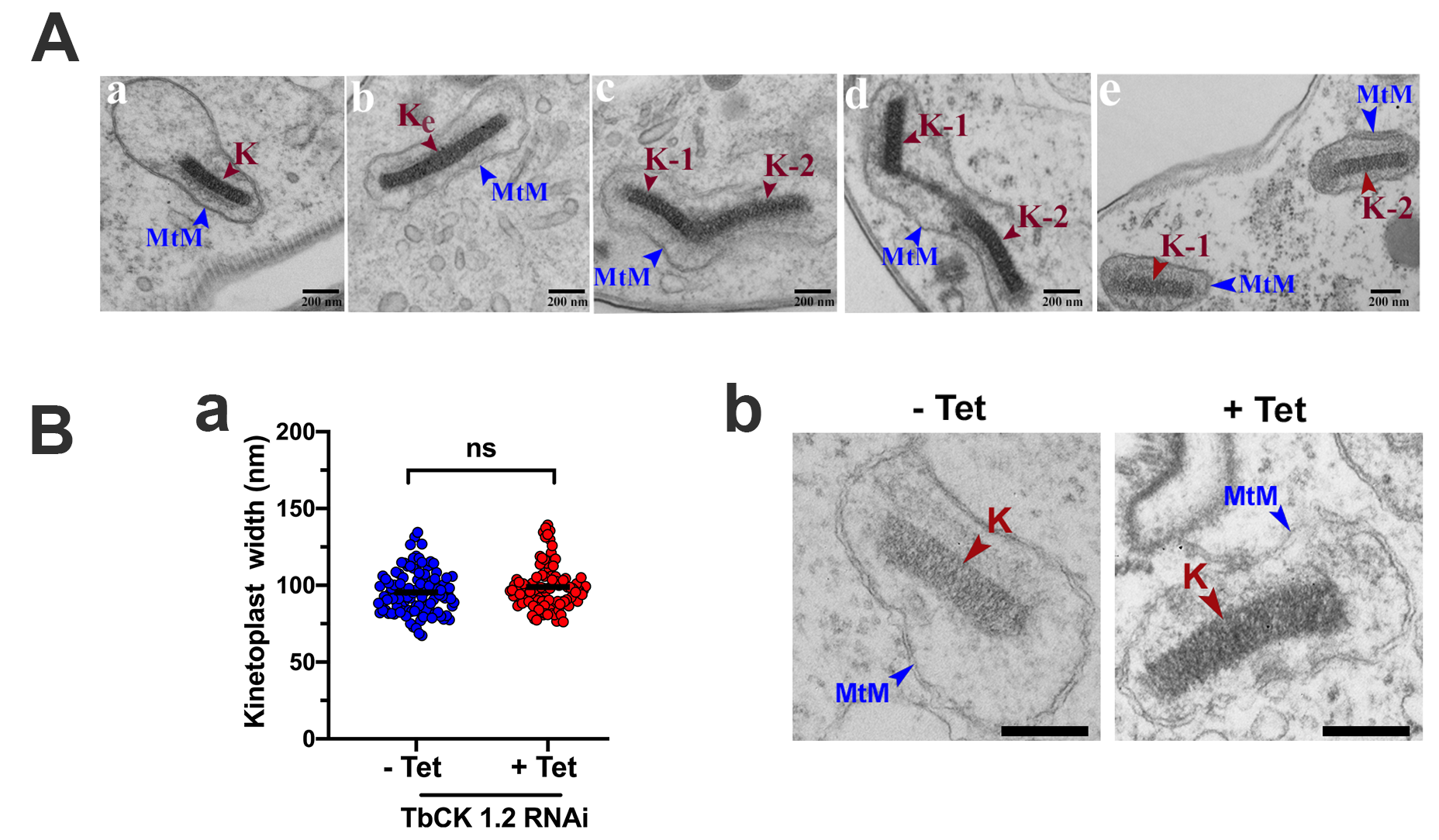

Supplement: S3 Fig — (A) Transmission electron microscopy images showing kinetoplast network duplication cycle in T. brucei. Panel a depicts a kinetoplast (K) of median length (~ 400 nm). Panel b is a representative image for elongated kinetoplast (Ke), whereas c and d show kinetoplasts at the scission and separation steps of division, respectively. Panel e shows two cleaved and separated kinetoplasts (K-1 and K-2), each surrounded by mitochondrial membranes (MtM). (B) Widths of kinetoplasts are not affected by knockdown of TbCK1.2. (a) A scatter plot showing the width of kinetoplast network in uninduced and induced population (24 h) of TbCK1.2 RNAi cell line. TEM images of kinetoplast were used for measurement of width, n = 100 for uninduced and induced population, ns; non-significant, Mann Whitney U test was used for statistical analysis. (b) Representative TEM images of kinetoplasts from uninduced and induced population of TbCK1.2 RNAi cell line. K; kinetoplast, MtM; mitochondrial membrane. Scale bar; 200 nm. (TIF) [file pone.0249908.s003.tif]

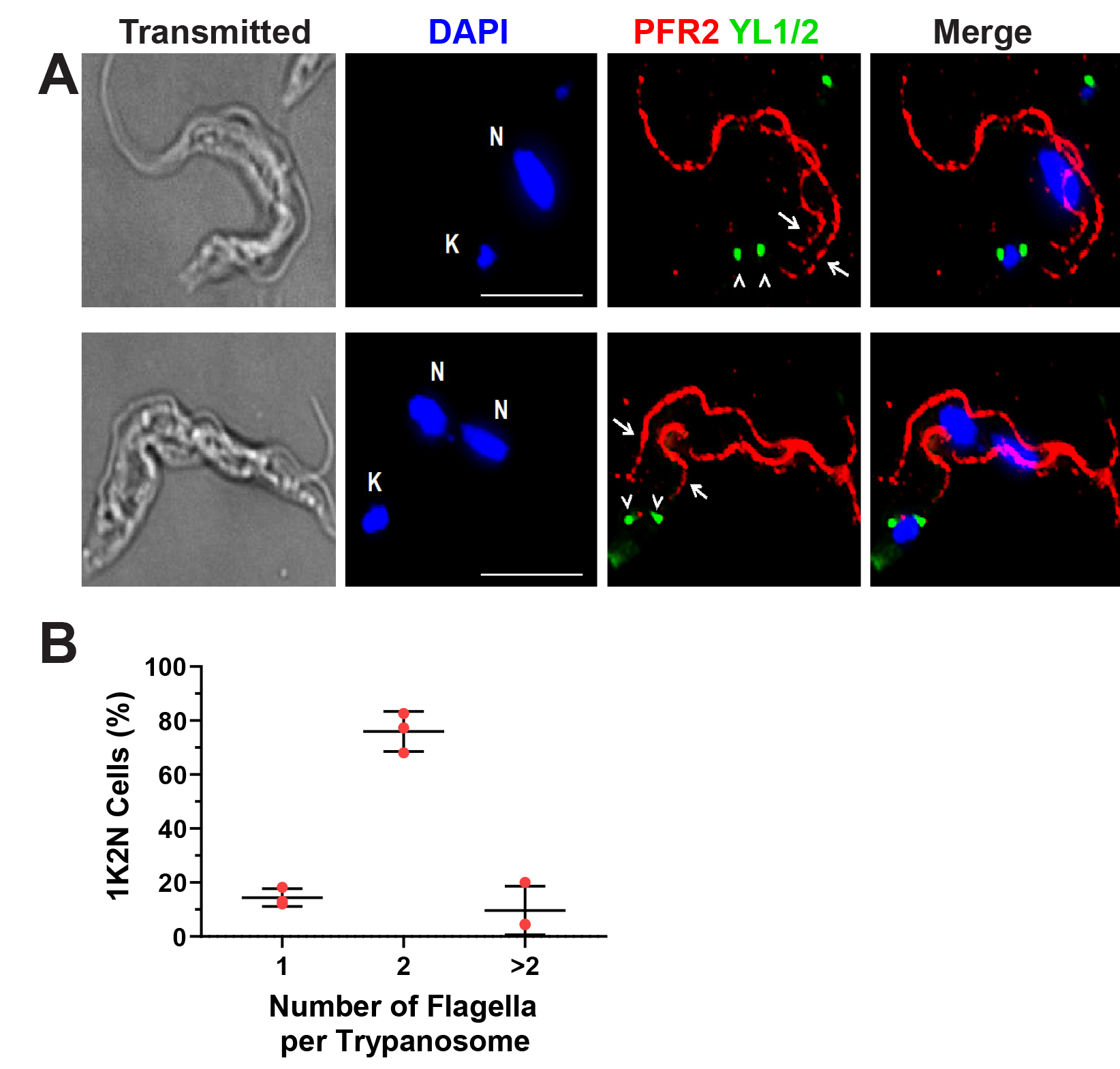

Supplement: S4 Fig — Knockdown of TbCK1.2 was induced with tetracycline. Cells were fixed with methanol and an antibody against TbRP2 (YL1/2) used to identify mature basal bodies. Flagella were detected with anti-PFR2 (against a paraflagellar rod protein). Kinetoplasts and nuclei were stained with DAPI. For all images the scale bar is 6 μm. (A) Representative staining of the TbRP2 (mature basal bodies) and PFR2 (flagellum) in TbCK1.2 RNAi cells cultured in the presence (+Tet) or absence of tetracycline (-Tet). Arrows indicate flagella, arrowheads indicate mature basal bodies. (B) The average percentage of 1K2N cells with the indicated number of flagella is shown, from three independent experiments (n = 96-130/experiment). (TIF) [file pone.0249908.s004.tif]

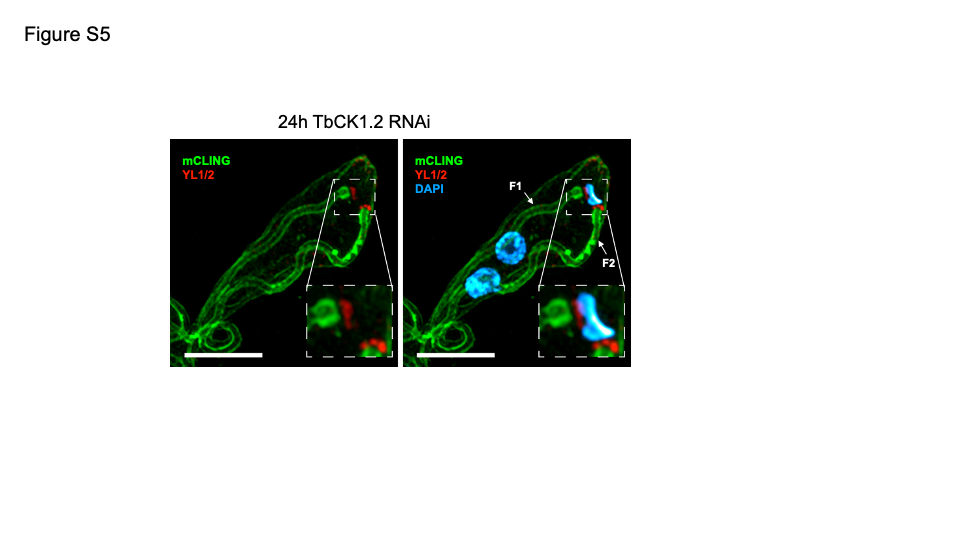

Supplement: S5 Fig — Membranes were stained with mCLING [5], basal bodies were labeled with YL1/2 antibody, and DNA was stained with DAPI. Maximum intensity projections of z-stack of images were acquired with an SR-SIM microscope. (TIF) [file pone.0249908.s005.tif]
