# Supplementary material for: Casein kinase TbCK1.2 regulates division of kinetoplast DNA, and movement of basal bodies in the African trypanosome
Source: PLoS One. 2021 Apr 16;16(4):e0249908. doi: 10.1371/journal.pone.0249908 (PMC8051774; doi:10.1371/journal.pone.0249908)
Supplement: S3 Table — Following a 24-h knockdown of TbCK1.2, phospho-peptides were harvested from uninduced and induced cells and phospho-peptides enriched over an IMAC column (see materials and methods). Phospho-peptide abundance was calculated in each sample using a labeled proteomics (SILAC) (n = 1) and label-free approach (spectral counting (SC)) (n = 2). Phospho-peptides identified with decreased abundance (at least 2-fold) in each phosphoproteomics strategy are listed. Phosphorylation sites are indicated in red (PhosphoRS [6] value >79%). * indicates the number of phospho-sites which could not be accurately assigned. The fold change in phospho-peptide abundance, as compared to the uninduced control, is shown. ~99 indicates that the phospho-peptide was only present in the control or induced population, preventing calculation of an abundance ratio. All listed peptides had a PEP value (probability that spectra-peptide match was incorrect) of 6% or less. N/A indicates that the exact phospho-isoform of the indicated peptide was not identified. A control experiment comparing the abundance ratio of phospho-peptides from uninduced cells grown in heavy or light SILAC medium was performed. Peptides that showed a 2-fold change in abundance in both the control and experimental group are not reported as TbCK1.2 pathway proteins. (DOCX) [file pone.0249908.s008.docx]

**Table S3**

| **Gene ID** | **Predicted Protein Product** | **Sequence** | **Fold Decrease** | |
| --- | --- | --- | --- | --- |
|  |  |  | **SILAC** | **SC** |
| Tb427.01.1880 | WD40 repeat-containing protein | SSQSAVTTSEVGGCSPQR* | 2.5 | 2 |
| Tb427.01.4280 | Hypothetical | VSASSTPQFSR | 3.1 | 4 |
| Tb427.01.4310 | FAZ Protein 2 | FDYLSDQRPR | 3.1 | 2 |
| Tb427.02.5810 | Hypothetical | GDVGDPAVSDGDGTDIGR | 3 | 3 |
| Tb427.03.1010 | Hypothetical | AVASLVTDEASEQAAAAPQNR | 8.7 | 3.5 |
| Tb427.03.3240 | Hypothetical | GNNSNSLNGSVNGPR | N/A | 2 |
| Tb427.03.3240 | Hypothetical | GNNSNSLNGSVNGPR | 2.6 | N/A |
| Tb427.03.3520 | Outer Mitochondrial Membrane Protein (POMP25) | EGSGFECSSGVLTQEER | 2.1 | ~99 |
| Tb427.03.3940 | RNA binding protein (DRBD11) | TPLNNESGPGTSSSGSHSSSSNVPVASLR** | N/A | 5 |
| Tb427.03.3940 | RNA binding protein (DRBD11) | TPLNNESGPGTSSSGSHSSSSNVPVASLR* | 2.5 | N/A |
| Tb427.03.5040 | Hypothetical | GLQDGVESDGCSTVFSHSGQR | 2.2 | N/A |
| Tb427.03.5040 | Hypothetical | GLQDGVESDGCSTVFSHSGQR* | N/A | 4 |
| Tb427.04.2750 | Hypothetical | LPTRGSQQPLDEDEDR | 2.2 | N/A |
| Tb427.04.2750 | Hypothetical | LPTRGSQQPLDEDEDR* | N/A | ~99 |
| Tb427.04.2920 | Hypothetical | NSVTFSDATETR | 2 | N/A |
| Tb427.04.2920 | Hypothetical | NSVTFSDATETR | N/A | 2.5 |
| Tb427.05.360 | ISG75 (invariant surface glycoprotein) | DDISIGEANAK | 2 | 2 |
| Tb427.05.3610 | adaptor complex protein 3 delta subunit 1  (AP3) | VVGATGSISNR | 2 | N/A |
| Tb427.05.3610 | adaptor complex protein 3 delta subunit 1  (AP3) | VVGATGSISNR | N/A | ~99 |
| Tb427.06.2840 | Rio2 Kinase | SIDSAINVAAQQR | ~99 | 4.3 |
| Tb427.06.3490 | ZFP-1 (Zinc finger binding protein 1) | SENSLSFSGSR | 5.3 | 2 |
| Tb427.06.4390 | Kinesin | DGTPSPNNTQNENLQR | 2 | N/A |
| Tb427.06.4390 | Kinesin | DGTPSPNNTQNENLQR | N/A | 2 |
| Tb427.06.4440 | RNA binding protein 42 | TGAVEKEPSCAEGK | 4.3 | ~99 |
| Tb427.07.2300 | TbNup132 (nucleoporin) | SEMETMSAPADPLSEK | 16.6 | N/A |
| Tb427.07.2300 | TbNup132 (nucleoporin) | SEMETMSAPADPLSEK** | N/A | ~99 |
| Tb427.07.3550 | Cytoskeleton Associated Protein | AAEGKPSTSEAESSDVGAAANTR | 3.2 | N/A |
| Tb427.07.3550 | Cytoskeleton Associated Protein | AAEGKPSTSEAESSDVGAAANTR** | N/A | 3.5 |
| Tb427.07.5030 | Hypothetical | GGSESEVYDTLNGSNSNNK | 2.4 | 2 |
| Tb427.08.3870 | SRP40, C-terminal domain containing protein | KPVAPDSSSDDDEPVR | 2.4 | N/A |
| Tb427.08.3870 | SRP40, C-terminal domain containing protein | KPVAPDSSSDDDEPVRKPLVK | N/A | 2.5 |
| Tb427.08.4400 | Hypothetical | NSVVAGTSDYNQR | 2.6 | 2 |
| Tb427.08.6660 | PFC1 (PFR component) | MMTMPDADGAADSNKGSLDTGSVPK* | 2.1 | 18 |
| Tb427.08.7080 | Hypothetical | SLDESTQHTISAPSK | 3.2 | 2.5 |
| Tb427.08.7760 | Hypothetical | TSATFLASPLQPVR* | 5.2 | N/A |
| Tb427.08.7760 | Hypothetical | TSATFLASPLQPVR | N/A | ~99 |
| Tb427.10.10280 | TbBBP268 | RHSFTASSEADAAVVK* | 3.7 | N/A |
| Tb427.10.10280 | TbBBP268 | RHSFTASSEADAAVVK | N/A | 5.5 |
| Tb427.10.11990 | RNA binding protein (Nip7 homolog) | TTGNNGSNDDDDGDDGEEQNSQQTYVFR* | 4 | 3 |
| Tb427.10.13800 | Hypothetical | VSSTTQPAAEAAVEKPADSGAPAVPDAEAETR** | N/A | ~99 |
| Tb427.10.13800 | Hypothetical | VSSTTQPAAEAAVEKPADSGAPAVPDAEAETR* | 2.3 | N/A |
| Tb427.10.14480 | Hypothetical | EALQGLGASEGSQTGR* | 2 | N/A |
| Tb427.10.14480 | Hypothetical | EALQGLGASEGSQTGR | N/A | ~99 |
| Tb427.10.14500 | Hypothetical | SATPPQGTIVMPGTVR | 8.2 | N/A |
| Tb427.10.14500 | Hypothetical | SATPPQGTIVMPGTVR | N/A | 2.9 |
| Tb427.10.15290 | tubulin binding cofactor c | SSMEGAGSVSSDEEADSAHIGR | 5.3 | N/A |
| Tb427.10.15290 | tubulin binding cofactor c | SSMEGAGSVSSDEEADSAHIGR | N/A | 2.5 |
| Tb427.10.15300 | S/T Protein Kinase | DQPFYSNGSGHGER | 2.7 | ~99 |
| Tb427.10.350 | TbBBP59 (Protein Kinase) | VSSAGSTPSVTAAR* | N/A | 3 |
| Tb427.10.350 | TbBBP59 (Protein Kinase) | VSSAGSTPSVTAAR* | 6.6 | N/A |
| Tb427.10.5200 | Hypothetical | SGGDDVDDIDGSGLAK | 3.9 | 2 |
| Tb427.10.5450 | NLP (ISWI complex) | EENSVNGDETNTTLPR | 2.4 | 2 |
| Tb427.10.970 | Hypothetical | SGPSSQDPFVCSTTAK* | 2.2 | N/A |
| Tb427.10.970 | Hypothetical | SGPSSQDPFVCSTTAK** | N/A | 3 |
| Tb427tmp.01.0680 | TbLRRP1 | LGRPPSTTNDDASHPAK | 4.0 | N/A |
| Tb427tmp.01.0680 | TbLRRP1 | LGRPPSTTNDDASHPAK* | N/A | 6 |
| Tb427tmp.01.3730; Tb427tmp.01.3720 | Hypothetical | SPSSDQLDVVK | 3.7 | N/A |
| Tb427tmp.01.3730; Tb427tmp.01.3720 | Hypothetical | SPSSDQLDVVK* | N/A | ~99 |
| Tb427tmp.01.4780 | Hypothetical | ANGDGCSDAEDLLR | 2.6 | 2 |
| Tb427tmp.01.6790 | Bilobe Protein | LDEEVPNIGQLSDGGGSPK | 4.7 | 2.3 |
| Tb427tmp.02.0990 | Dpy-30 motif/AAA domain containing protein | SRQSLPTVIDLGTQAEK | 4.5 | 2.2 |
| Tb427tmp.211.1070 | ZC3H28 | QQGPAGSQVDEHEEDGDLEDSR | 3.0 | 2 |
| Tb427tmp.211.2360; Tb427tmp.211.2410 | protein kinase A catalytic subunit isoform 1/2 | SPGDTSNFESYPESGDK | 2.1 | N/A |
| Tb427tmp.211.2360 | protein kinase A catalytic subunit isoform 1 | SPGDTSNFESYPESGDKR | N/A | 2 |
| Tb427tmp.244.2660 | CHAT domain containing protein | STAQEADVDEKPQCLANR | 3.7 | 4 |
