# Supplementary material for: Casein kinase TbCK1.2 regulates division of kinetoplast DNA, and movement of basal bodies in the African trypanosome
Source: PLoS One. 2021 Apr 16;16(4):e0249908. doi: 10.1371/journal.pone.0249908 (PMC8051774; doi:10.1371/journal.pone.0249908)
Supplement: S4 Table — Following a 24-h knockdown of TbCK1.2, phospho-peptides were harvested from uninduced and induced cells and phospho-peptides enriched over an IMAC column (see materials and methods). Phospho-peptide abundance was calculated in each sample using a labeled proteomics (SILAC) (n = 1) and label-free approach (spectral counting (SC)) (n = 2). Phospho-peptides identified with increased abundance (at least 2-fold) in each phosphoproteomics strategy are listed. Phosphorylation sites are indicated in red (PhosphoRS [6] value >79%). * indicates the number of phospho-sites which could not be accurately assigned. The fold change in phospho-peptide abundance, as compared to the uninduced control, is shown. ~99 indicates that the phospho-peptide was only present in the control or induced population, preventing calculation of an abundance ratio. All listed peptides had a PEP value (probability that spectra-peptide match was incorrect) of 6% or less. N/A indicates that the exact phospho-isoform of the indicated peptide was not identified. A control experiment comparing the abundance ratio of phospho-peptides from uninduced cells grown in heavy or light SILAC medium was performed. Peptides that showed a 2-fold change in abundance in both the control and experimental group are not reported as putative TbCK1.2 effectors. (DOCX) [file pone.0249908.s009.docx]

**Table S4**

| **Gene ID** | **Predicted Protein Product** | **Sequence** | **Fold Increase** | |
| --- | --- | --- | --- | --- |
|  |  |  | **SILAC** | **SC** |
| Tb427.01.2100 | Calpain-like cysteine peptidase | ANKSEGESVTKDGSDGHAEETSPVQSPEGEVGER** | ~99 | 3 |
| Tb427.01.4310 | FAZ Protein 2 | SSGTALPAGAGVSEMMHTCR | 3.5 | ~99 |
| Tb427.02.1820 | Protein Kinase (SNF1/CBL-interacting) | SPHSATTAAEASITSFAK* | 2.1 | 4 |
| Tb427.02.5760 | Flagellar Member 8 | KSASPSELNSPVMK* | N/A | 5 |
| Tb427.02.5760 | Flagellar Member 8 | KSASPSELNSPVMK | 2 | N/A |
| Tb427.02.5760 | Flagellar Member 8 | SDLPSSPSSPLCIK | N/A | 3 |
| Tb427.02.5760 | Flagellar Member 8 | SDLPSSPSSPLCIK | 2.1 | N/A |
| Tb427.03.1010 | Hypothetical | GAADVNENPTSSATPR* | N/A | 4 |
| Tb427.03.1010 | Hypothetical | GAADVNENPTSSATPR | ~99 | N/A |
| Tb427.03.1010 | Hypothetical | GGSVESAATRPSGGGAALTQDAVDAGGSAADSNAR | N/A | 2.7 |
| Tb427.03.1010 | Hypothetical | GGSVESAATRPSGGGAALTQDAVDAGGSAADSNAR | ~99 | N/A |
| Tb427.03.3800 | Hypothetical | EHPPSFQSPTPTVEGPLVSPR | 4 | 3 |
| Tb427.03.3880 | Hypothetical | EPMSPLPTQPTSVPSVASLK | ~99 | ~99 |
| Tb427.03.3940 | RNA-binding protein (DRBD11) | SLGISGHGSAR | 2.9 | 2 |
| Tb427.03.4180 | Hypothetical | VSSPLPPIDASEHGSPR* | 11.1 | ~99 |
| Tb427.03.4270 | Hypothetical | FPALSGSVVR | N/A | 0 |
| Tb427.03.4270 | Hypothetical | FPALSGSVVR | 2.1 | N/A |
| Tb427.03.4270 | Hypothetical | LTESLQNVNDR | 3.2 | 2 |
| Tb427.03.4710 | Hypothetical | LSETSSSSVAASR** | ~99 | ~99 |
| Tb427.03.4710 | Hypothetical | SHSSNVESGCTSR | N/A | ~99 |
| Tb427.03.4710 | Hypothetical | SHSSNVESGCTSR* | 2.7 | N/A |
| Tb427.03.4970 | Hypothetical | GQTGAGGSGPGPSGAVESDLLQK* | ~99 | 4 |
| Tb427.03.5020 | Flagellar Member 6 | RPNADPDEKSDSGTHSEGEHTMEK** | N/A | ~99 |
| Tb427.03.5020 | Flagellar Member 6 | RPNADPDEKSDSGTHSEGEHTMEK* | ~99 | N/A |
| Tb427.04.1700 | Protein Kinase (Tau-tubulin Kinase) | GHSASPEPPPPFQR | 2.2 | ~99 |
| Tb427.04.2370 | Hypothetical | HTTNSSFSSNIGSR* | 5.7 | ~99 |
| Tb427.04.2820 | Hypothetical | STSTTASHALQQGGAETSDQSR** | 2.4 | N/A |
| Tb427.04.2820 | Hypothetical | STSTTASHALQQGGAETSDQSR* | N/A | ~99 |
| Tb427.04.3140 | SBDS protein C-terminal domain containing protein | SVGGGGGSHQTGSSSNPTQCLNNNNK* | 2.1 | ~99 |
| Tb427.04.3330 | EF-hand domain pair | TVGDSSKNASTSSVTNAVK | 5.9 | N/A |
| Tb427.04.3330 | EF-hand domain pair | TVGDSSKNASTSSVTNAVK** | N/A | ~99 |
| Tb427.04.4280 | Hypothetical | VLCSEPPTPPCEQK | ~99 | 2.5 |
| Tb427.05.2620 | Hypothetical | RPVSSPIACGHGSR | 3 | ~99 |
| Tb427.05.3030 | IFT122B | LDGTTTSLQLTNPSK** | N/A | 0 |
| Tb427.05.3030 | IFT122B | LDGTTTSLQLTNPSK* | 4.5 | N/A |
| Tb427.05.3030 | IFT122B | VGHGVGPAGGGAGGVGGTTR | 2.7 | 4 |
| Tb427.06.1180 | Hypothetical | RVESDPSQLADSPEPQKPPR | 2.1 | 2 |
| Tb427.06.1920 | Hypothetical | NSQTLQDGMGSSSR* | ~99 | 3.3 |
| Tb427.06.2860 | Hypothetical | GSTISCSSPQRPQAVVNELHR* | N/A | 8 |
| Tb427.06.2860 | Hypothetical | GSTISCSSPQRPQAVVNELHR* | 2.4 | N/A |
| Tb427.06.4390 | KIF3/5 Heavy Chain | GPSPFDAAR | ~99 | 3 |
| Tb427.06.5010 | Hypothetical | DASEHLPALPSAR | 3.1 | N/A |
| Tb427.06.5010 | Hypothetical | DASEHLPALPSAR* | N/A | 3 |
| Tb427.06.620 | Hypothetical | TPLSPVSSR | 3.6 | ~99 |
| Tb427.06.870 | myotubularin | SLPFLDER | 3.2 | ~99 |
| Tb427.07.1420 | Hypothetical | RASFAFGDSCAASPR | 2.6 | ~99 |
| Tb427.07.2320 | Hypothetical | VLQPLSSGSPSPR* | 2.1 | 3 |
| Tb427.07.2660 | ZC3H20 | SVTLGDASVTTQPAVVR | 4.5 | ~99 |
| Tb427.07.3130 | Hypothetical | EVSQRPVGVSPGDAATDTSPLK | 3.6 | N/A |
| Tb427.07.3130 | Hypothetical | EVSQRPVGVSPGDAATDTSPLK** | N/A | ~99 |
| Tb427.07.3130 | Hypothetical | TATETATEGYSQPPSVTVYPHVNR | 3.2 | N/A |
| Tb427.07.3130 | Hypothetical | TATETATEGYSQPPSVTVYPHVNR | N/A | ~99 |
| Tb427.07.3700 | Hypothetical | AASFESPSDDTLR | 3.9 | 2 |
| Tb427.07.3790 | ras-like small GTPase | SNVSSPLLSK | 7.2 | 2 |
| Tb427.07.4410 | Hypothetical | SSFNAVETHR | ~99 | 2 |
| Tb427.07.4410 | Hypothetical | SSVSPVSSTTTATETHHPETTSSSTR** | N/A | ~99 |
| Tb427.07.4410 | Hypothetical | SSVSPVSSTTTATETHHPETTSSSTR* | 2.4 | N/A |
| Tb427.07.4500 | PX domain containing protein | EQPQPVAVVDSTPPPAPK* | 5.1 | 2 |
| Tb427.07.4870 | Hypothetical | MASAASTDIR | 2 | N/A |
| Tb427.07.4870 | Hypothetical | MASAASTDIR* | N/A | ~99 |
| Tb427.07.5140 | Hypothetical | DAEAVLSPTSDPDAK* | ~99 | ~99 |
| Tb427.07.5180; Tb427.07.5170 | 60S ribosomal protein L23a | LSASYDALDTANK | 7.2 | ~99 |
| Tb427.07.6790 | Hypothetical | NGASIQPSCDETTPDKVQNALTESSVVSR* | 4.5 | ~99 |
| Tb427.07.6950 | Hypothetical | VGHVPGVQLSPK | ~99 | ~99 |
| Tb427.07.7000 | Hypothetical | SQLEVQAPAR | 2.6 | ~99 |
| Tb427.07.7250 | Ankyrin repeats (3 copies) | HSISSQQR | 2 | ~99 |
| Tb427.08.2640 | ubiquitin-activating enzyme e1 (UBA1) | ATTECAQGDNSPTGASSSLR | ~99 | 2 |
| Tb427.08.3180 | DUF3250 | SSSFTLVPR | 2 | ~99 |
| Tb427.08.3590 | Hypothetical | TGSIFSGEK | 2.2 | 2 |
| Tb427.08.4400 | Hypothetical | GSCFTDSVTNGIVPIGGGK* | 4.9 | 2.5 |
| Tb427.08.4780 | Flagellar Member 3 | DQTPSLQDLLR | 3.4 | N/A |
| Tb427.08.6050 | Hypothetical | AVISPQEKPLTSSSSGEALGGSGNEVK* | ~99 | ~99 |
| Tb427.08.6370 | cytoskeleton associated protein | EEGSLSPYLR | 6.3 | ~99 |
| Tb427.08.6660 | PFR component 1 | ITLDKSQISK | 4.5 | 5 |
| Tb427.08.6950; Tb427.04.5370 | dynein light chain 2B | MEFNASTTNER | 3.6 | ~99 |
| Tb427.08.7820 | DNA-binding domain containing protein | IASPPPPSR | ~99 | ~99 |
| Tb427.08.7850 | Hypothetical | AGPISHVEGSPSR | ~99 | ~99 |
| Tb427.08.790 | Hypothetical | KGSCACSTTNISDTNAAQNSR* | N/A | ~99 |
| Tb427.08.790 | Hypothetical | KGSCACSTTNISDTNAAQNSR* | ~99 | N/A |
| Tb427.10.11880 | Hypothetical | ANASTESAGVSGEDALER | N/A | 4 |
| Tb427.10.11880 | Hypothetical | ANASTESAGVSGEDALER | 2.6 | N/A |
| Tb427.10.12950 | BBP110 | EESHCPGASAAPSSR | 2.5 | ~99 |
| Tb427.10.13780 | glycogen synthase kinase 3 | STGSLVAIK | ~99 | ~99 |
| Tb427.10.14300 | MEKK-related kinase 1 (MRK1) | FNDASESDPNDDDDDNSSTSTAGPPGSTR** | ~99 | ~99 |
| Tb427.10.14490 | Hypothetical | TNGSGSTGSGEAAAGEPNAQK | ~99 | ~99 |
| Tb427.10.1810 | RING-H2 zinc finger | MEEAAAEGMPLSQEQGEQK | 2.1 | 3 |
| Tb427.10.3500 | RNA-binding protein | TSQNDGAIVPLLAEDVEK* | 2.6 | 3 |
| Tb427.10.4440 | predicted SAP domain protein | ASTGSVSESGHVSGLK | N/A | ~99 |
| Tb427.10.4440 | predicted SAP domain protein | ASTGSVSESGHVSGLK* | 4 | N/A |
| Tb427.10.4860 | Hypothetical | SCTPPPGDSDVSR | ~99 | ~99 |
| Tb427.10.5350 | dynein heavy chain | SELQASQVGASSETAVVR* | 2.1 | ~99 |
| Tb427.10.5880 | Proteophosphoglycan | SASLDSSVTAK | ~99 | ~99 |
| Tb427.10.5880 | Proteophosphoglycan | SNISSTCLTPGR* | 2.6 | 2 |
| Tb427.10.6410 | mismatch repair protein (MSH6) | TSEGPTQEFTQASGTQCGSK* | ~99 | ~99 |
| Tb427.10.6580 | hypothetical protein | HASLPSNSTPVK** | N/A | 2 |
| Tb427.10.6580 | hypothetical protein | HASLPSNSTPVK* | 3.7 | N/A |
| Tb427.10.7230 | Flagellar Member 1 | DGTASTPTQERHSTLGEETEGPMTVSSR** | 2.1 | ~99 |
| Tb427.10.9330 | hypothetical protein | ASGEVNAESNVHSPASVTAK | 3.7 | ~99 |
| Tb427.10.9330 | hypothetical protein | VEGDGSPELLATR | 2 | ~99 |
| Tb427.10.9700 | predicted C2 domain protein | SYASSADAFSSSAQR* | 2.6 | ~99 |
| Tb427.10.9700 | predicted C2 domain protein | TTASTACTSSGYNTAR | 2.9 | ~99 |
| Tb427.10.9770 | gamma-tubulin complex component 2 (GCP2) | GAFAPPPPLQAPQTAAATVSSPTR* | 2.5 | ~100 |
| Tb427tmp.01.0680 | TbLRRP1 | SASAVELYSLR | 3.1 | 3 |
| Tb427tmp.01.0920 | ADP-ribosylation factor GTPase activating protein | GPSQGNFQAPAVDAK | 2.4 | 2 |
| Tb427tmp.01.1050 | FAZ Protein 20 | MLNNIPSQR | 2.2 | ~99 |
| Tb427tmp.01.2330 | eukaryotic translation initiation factor 4 γ | CSQSTNDLTR | 2.2 | 3 |
| Tb427tmp.01.2430 | BBP590 | VSGASTVSGMQTAASSSSSSAR | ~99 | ~99 |
| Tb427tmp.01.3960 | BILBO1 | VTPNGSLSMQGALAPYNGSR | 2.3 | 2 |
| Tb427tmp.01.4320 | kinetoplastid-specific phospho-protein phosphatase | EGSLASDGLVSHR | 2.4 | 2 |
| Tb427tmp.01.4400 | hypothetical protein | KDSSPPRPPFR | 3.4 | ~99 |
| Tb427tmp.01.4400 | hypothetical protein | SPSTMSQPQQLEYETR | N/A | ~99 |
| Tb427tmp.01.4400 | hypothetical protein | SPSTMSQPQQLEYETR* | 3.1 | N/A |
| Tb427tmp.01.4480 | PH domain containing protein | LSKPQQPSNSSGGDSK | ~99 | 2.5 |
| Tb427tmp.01.4920 | hypothetical protein | GGTSGDDSATPSTLDDDESRGSGEEK | N/A | ~99 |
| Tb427tmp.01.4920 | hypothetical protein | GGTSGDDSATPSTLDDDESRGSGEEK | ~99 | N/A |
| Tb427tmp.01.6770 | hypothetical protein | KNADDYSETSGTALPGEAGEK* | 4 | ~99 |
| Tb427tmp.01.6770 | hypothetical protein | SSSEIVASTGGGTDTHRDSGSSGNNGAAPNDNEK* | ~99 | 10 |
| Tb427tmp.01.6790 | hypothetical protein | STSALLSSLGGK | 2 | 2.3 |
| Tb427tmp.01.6900 | hypothetical protein | LEGTPTSDSNAPR | 3.9 | ~99 |
| Tb427tmp.01.8190 | hypothetical protein | TEAVPLSR | 3.7 | 2 |
| Tb427tmp.01.8770 | leucine-rich repeat protein (LRRP) | SAITGPVAAGPPECESIK | 5 | ~99 |
| Tb427tmp.02.0350 | Outer Mitochondrial Membrane Protein (POMP12) | DGSHTTNDSTDCSTVTSAR** | N/A | 2 |
| Tb427tmp.02.0350 | Outer Mitochondrial Membrane Protein (POMP12) | DGSHTTNDSTDCSTVTSAR* | 2.3 | N/A |
| Tb427tmp.02.1420 | hypothetical protein | YSPLHFCSSK | 3.1 | 4 |
| Tb427tmp.02.1600 | ubiquitin-like protein | FEGTQTPQQTR | 3.1 | ~99 |
| Tb427tmp.02.4290 | hypothetical protein | GSVATSASNQEATCAANPSGIDSSR* | N/A | 2.3 |
| Tb427tmp.02.4290 | hypothetical protein | GSVATSASNQEATCAANPSGIDSSR | 3.7 | N/A |
| Tb427tmp.02.4290 | hypothetical protein | GVSTHSAGCQSESGASSVVSSTEHVQNNK** | N/A | ~99 |
| Tb427tmp.02.4290 | hypothetical protein | GVSTHSAGCQSESGASSVVSSTEHVQNNK | 2.4 | N/A |
| Tb427tmp.02.4640 | tubulin-tyrosine ligase-like protein | EESMGGAAGTGHISEDGGSK | 8.3 | 2 |
| Tb427tmp.02.4950 | cytoplasmic translation associated protein | HLSSADLFK* | N/A | ~99 |
| Tb427tmp.02.4950 | cytoplasmic translation associated protein | HLSSADLFK | ~99 | N/A |
| Tb427tmp.160.1440 | EB1-like C-terminal motif containing protein | SGSRGESSSEGDSAVAAAALLK* | 2.8 | 2 |
| Tb427tmp.160.1440 | EB1-like C-terminal motif containing protein | TNNAGSSSGVLSGSGDISER | 3 | 2 |
| Tb427tmp.160.2980 | GTPase activating protein | SASVTMEGESVR | ~99 | ~99 |
| Tb427tmp.160.3020 | hypothetical protein | SAGNDDKSHPPTQGGFR | 2.2 | 2 |
| Tb427tmp.160.3990 | cation transporter | EASPGSATLLDSPAATGMSTR | 8.5 | ~99 |
| Tb427tmp.160.4700 | peroxisomal membrane protein (Pex16) | DRSSETVGEDADFSEAGK | ~99 | 2 |
| Tb427tmp.211.3890 | hypothetical protein | DHASPAEGSGSNVTVVSGVCSAER | N/A | 3 |
| Tb427tmp.211.3890 | hypothetical protein | DHASPAEGSGSNVTVVSGVCSAER* | 3.3 | N/A |
| Tb427tmp.211.4840 | mismatch repair protein (PMS1) | TSSPDATASPTSTTNR | N/A | ~99 |
| Tb427tmp.211.4840 | mismatch repair protein (PMS1) | TSSPDATASPTSTTNR* | 3.2 | N/A |
| Tb427tmp.39.0006 | translation initiation factor eIF2B delta subunit | SPGNWSPLSHGSR | 2.5 | 4 |
| Tb427tmp.55.0019 | hypothetical protein | SKSPPAPLVPR | 5.6 | 3 |
