# Supplementary material for: Casein kinase TbCK1.2 regulates division of kinetoplast DNA, and movement of basal bodies in the African trypanosome
Source: PLoS One. 2021 Apr 16;16(4):e0249908. doi: 10.1371/journal.pone.0249908 (PMC8051774; doi:10.1371/journal.pone.0249908)
Supplement: S5 Table — “TbCK1.2-Pathway Proteins” (S4 and S5 Tables) were re-analyzed in search for mitochondrial proteins as follows. (A) Gene IDs for TbCK1.2-pathway proteins were compared to proteins that localize to the mitochondrion in the TrypTag database. (B) Gene IDs for TbCK1.2-pathway proteins were compared to two mitochondrial proteomes (combined) containing 1730 proteins [7, 8] available in TryTripDB (release 41) [9]. Polypeptides found in both data sets were filtered by eliminating proteins in glycosome or nucleus proteomes [10, 11], resulting in 21 proteins. (Proteins are imported post-translationally into both nuclei and glycosomes [12, 13]). (DOCX) [file pone.0249908.s010.docx]

**Table S5**


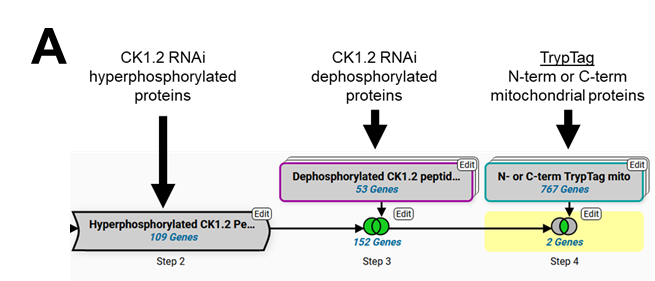


| **Gene ID** | **Product Description** |
| --- | --- |
| Tb927.6.4390 | Kinesin heavy chain 1, putative |
| Tb927.8.7820 | 'Cold-shock' DNA-binding domain containing protein, putative |


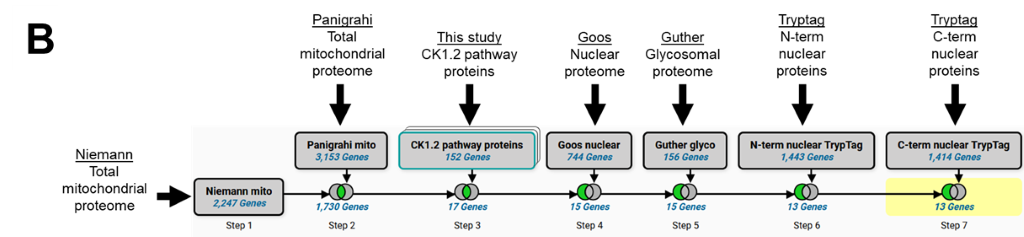


| **Gene ID** | **Product Description** | **Location (TrypTag)** |
| --- | --- | --- |
| Tb927.1.2100 | Calpain-like protein 1.1 | Paraflagellar rod |
| Tb927.1.4310 | Flagellum attachment zone protein 2 | Flagellum attachment zone |
| Tb927.3.1010 | hypothetical protein, conserved | Not determined |
| Tb927.3.3520 | Present in the outer mitochondrial membrane proteome 25 | Not determined |
| Tb927.7.3550 | cytoskeleton associated protein, putative | Cortical cytoskeleton |
| Tb927.8.4780 | Flagellar Member 3 | Flagellum attachment zone, cytoplasm (reticulated) |
| Tb927.8.6660 | paraflagellar rod component, putative | Paraflagellar rod |
| Tb927.10.10280 | Microtubule-associated repetitive protein 2 | Not determined |
| Tb927.10.14490 | hypothetical protein, conserved | Flagellum, cytoplasm |
| Tb927.10.14500 | hypothetical protein, conserved | Flagellum |
| Tb927.11.10560 | eukaryotic translation initiation factor 4 gamma 4 | Cytoplasm |
| Tb927.11.17000 | AIR9-like protein | Not determined |
| Tb927.11.2750 | Present in the outer mitochondrial membrane proteome 12 | Cytoplasm |
