# Supplementary material for: Casein kinase TbCK1.2 regulates division of kinetoplast DNA, and movement of basal bodies in the African trypanosome
Source: PLoS One. 2021 Apr 16;16(4):e0249908. doi: 10.1371/journal.pone.0249908 (PMC8051774; doi:10.1371/journal.pone.0249908)
Supplement: S1 File — (DOCX) [file pone.0249908.s011.docx]

**Supplemental Experimental Procedures**

**Generation of TbCK1.2 RNAi cell lines**

*TbCK1.2 RNAi line*: A p2T7 RNAi construct targeting TbCK1.2 [14] (provided by Dr. Mick Urbaniak, Lancaster University) was linearized with Not1-HF (New England Biolabs, Ipswich, MA) and electroporated into SM trypanosomes [15]. Following transfection, trypanosomes were added to HMI-9 medium (20 ml) and incubated for 24 hours. Subsequently, the culture was diluted serially (1:10, 1:~99, 1:~990) and each dilution plated (1 ml/well) in a 24-well plate. Stable transfectant clones were selected in the presence of G418 (6.5 µg/ml) and hygromycin B (5 µg/ml).

*V5-TbCK1.2 RNAi line*: A blasticidin (bla)/V5 plasmid (provided by Dr. Christian Tschudi, Yale University) was used as a template to amplify a bla/V5 tagging cassette flanked by sequences specific to TbCK1.2 in order to integrate a V5 epitope tag at the N-terminus of an endogenous TbCK1.2 allele [16]. The forward and reverse primer sequences were as follows: cagcggtgacagcggcaataatccaaccaaaatcaaacaaaaaaaaagagaagaaagagaatcaaaaacagaaactgtcggttataaacacccgggATGGCCAAGCCTTTGTCTCAAGAAG (forward) and GAATATTTGTCCCCCGGAATATTTCACCAAAC GAACCGGAACCAATTTTTTGCCCGATGCGGAATCGGTTTCCCACACGAAGCTCTACGCTcccgggCGTAGAATCGAGACCGAGGAGAGGGTTAG (reverse) (lower case letters indicate a linker between sequences corresponding to the plasmid template or to TbCK1.2). Primer design and PCR amplification were performed according to [17] before transfection into TbCK1.2 RNAi trypanosomes (4 x 10^7^) by nucleofection [18, 19]. After serial dilutions (see above), stable clones were selected in G418 (6.5 µg/ml), blasticidin (10 µg/ml), and hygromycin B (5 µg/ml), after serial dilution (as described above).

**Antibodies**

Antibodies were used at the following dilutions: YL1/2 [20] (EMD Millipore; Billerica, MA) targeting TbRP2 at 1:~990; anti-TbSAS6 [21] provided by Dr. Ziyin Li (University of Texas Health Sciences Center) at 1:500; 20H5 [22] (EMD Millipore) at 1:500; anti-PFR2 [23] at 1:500; anti-V5 (Cell Signaling; Danvers, MA) at 1:250 for immunofluorescence (IF) and 1:2000 for western blotting (WB). Anti-HA antibody was used at 1:250 for immunofluorescence. Secondary antibodies for IF were conjugated to either AlexaFluor-488 (AF-488) or AF-594 and used at 1:3000. For WB an anti-rabbit secondary antibody conjugated to alkaline phosphatase (Bio-Rad) was used at a dilution 1:3000.

**LC-MS/MS of labeled and unlabeled trypanosome peptides**

*Preparation of labeled peptides***:** A tetracycline-inducible TbCK1.2 RNAi line was cultured for five days in HMI-9 (17 doublings) medium modified for SILAC [24, 25]. Induced (light medium) and uninduced (heavy medium) trypanosomes (3 x 10^7^ cells per sample) were combined and processed as described [26].

*Label-free Peptide Preparation:* Trypanosomes with a tetracycline-inducible RNAi construct (5 x 10^4^ cells/ml) were incubated in the presence or absence of tetracycline in HMI-9 medium for 24 hours. Trypanosomes (6 x 10^7^ per sample) were processed as described for cells grown in SILAC medium, except that the uninduced and induced samples were prepared separately (n = 2).

*Phospho-peptide enrichment and LC-MS/MS analysis:* Phospho-peptides were enriched by metal affinity chromatography (IMAC), desalted twice with ZipTip U-C18 (Merck Millipore Ltd.), and analyzed as described [26].

An inclusion list [27] was used during analysis of the second label-free experiment. The list consisted of 17 unique peptides (which demonstrated the greatest decrease in phospho-peptide abundance in the first label-free experiment). A dephosphorylated version and multiple phospho-isoforms of each peptide were included (105 sequences total). During the survey scan, precursor ions matching the mass to charge ratios in the inclusion list were isolated first for MS/MS, before analyzing the most abundant ions.

**Western blotting**

Total cell lysate from trypanosomes (8 x 10^5^ per sample) was used for western blotting [19]. Proteins were separated on a TGX Stain-Free™ FastCast™ 12% acrylamide gel (Bio-Rad; Hercules, CA), and the Stain-Free gel activated for 5 minutes [4] before transfer of proteins to a PVDF membrane. Normalization of western blots was performed with Image Lab™ Software. Briefly, the Stain-Free blot image (detected under UV light) was used to estimate total protein in each lane. Image Lab™ was used to normalize band intensity of V5-TbCK1.2 to total protein per lane. Western blots were performed in triplicate, and the normalized band intensities obtained for either the uninduced or induced samples were averaged.

1. Schneider A, Ochsenreiter T: **Failure is not an option - mitochondrial genome segregation in trypanosomes**. *J Cell Sci* 2018, **131**(18).

2. Mensa-Wilmot K, Hoffman B, Wiedeman J, Sullenberger C, Sharma A: **Kinetoplast Division Factors in a Trypanosome**. *Trends Parasitol* 2019, **35**(2):119-128.

3. Gurtler A, Kunz N, Gomolka M, Hornhardt S, Friedl AA, McDonald K, Kohn JE, Posch A: **Stain-Free technology as a normalization tool in Western blot analysis**. *Analytical biochemistry* 2013, **433**(2):105-111.

4. Gilda JE, Gomes AV: **Western blotting using in-gel protein labeling as a normalization control: stain-free technology**. *Methods Mol Biol* 2015, **1295**:381-391.

5. Wiedeman J, Mensa-Wilmot K: **A fixable probe for visualizing flagella and plasma membranes of the African trypanosome**. *PLoS One* 2018, **13**(5):e0197541.

6. Taus T, Kocher T, Pichler P, Paschke C, Schmidt A, Henrich C, Mechtler K: **Universal and confident phosphorylation site localization using phosphoRS**. *Journal of proteome research* 2011, **10**(12):5354-5362.

7. Panigrahi AK, Ogata Y, Zikova A, Anupama A, Dalley RA, Acestor N, Myler PJ, Stuart KD: **A comprehensive analysis of Trypanosoma brucei mitochondrial proteome**. *Proteomics* 2009, **9**(2):434-450.

8. Niemann M, Wiese S, Mani J, Chanfon A, Jackson C, Meisinger C, Warscheid B, Schneider A: **Mitochondrial outer membrane proteome of Trypanosoma brucei reveals novel factors required to maintain mitochondrial morphology**. *Molecular & cellular proteomics : MCP* 2013, **12**(2):515-528.

9. Aslett M, Aurrecoechea C, Berriman M, Brestelli J, Brunk BP, Carrington M, Depledge DP, Fischer S, Gajria B, Gao X *et al*: **TriTrypDB: a functional genomic resource for the Trypanosomatidae**. *Nucleic acids research* 2010, **38**(Database issue):D457-462.

10. Guther ML, Urbaniak MD, Tavendale A, Prescott A, Ferguson MA: **High-confidence glycosome proteome for procyclic form Trypanosoma brucei by epitope-tag organelle enrichment and SILAC proteomics**. *Journal of proteome research* 2014, **13**(6):2796-2806.

11. Goos C, Dejung M, Janzen CJ, Butter F, Kramer S: **The nuclear proteome of Trypanosoma brucei**. *PLoS One* 2017, **12**(7):e0181884.

12. Guerra-Giraldez C, Quijada L, Clayton CE: **Compartmentation of enzymes in a microbody, the glycosome, is essential in Trypanosoma brucei**. *J Cell Sci* 2002, **115**(Pt 13):2651-2658.

13. Bauer S, Morris MT: **Glycosome biogenesis in trypanosomes and the de novo dilemma**. *PLoS neglected tropical diseases* 2017, **11**(4):e0005333.

14. Urbaniak MD: **Casein kinase 1 isoform 2 is essential for bloodstream form Trypanosoma brucei**. *Molecular and biochemical parasitology* 2009, **166**(2):183-185.

15. Subramanya S, Mensa-Wilmot K: **Regulated cleavage of intracellular glycosylphosphatidylinositol in a trypanosome. Peroxisome-to-endoplasmic reticulum translocation of a phospholipase C**. *FEBS J* 2006, **273**(10):2110-2126.

16. Shen S, Arhin GK, Ullu E, Tschudi C: **In vivo epitope tagging of Trypanosoma brucei genes using a one step PCR-based strategy**. *Molecular and biochemical parasitology* 2001, **113**(1):171-173.

17. Oberholzer M, Morand S, Kunz S, Seebeck T: **A vector series for rapid PCR-mediated C-terminal in situ tagging of Trypanosoma brucei genes**. *Molecular and biochemical parasitology* 2006, **145**(1):117-120.

18. Burkard G, Fragoso CM, Roditi I: **Highly efficient stable transformation of bloodstream forms of Trypanosoma brucei**. *Molecular and biochemical parasitology* 2007, **153**(2):220-223.

19. Guyett PJ, Xia S, Swinney DC, Pollastri MP, Mensa-Wilmot K: **Glycogen Synthase Kinase 3beta Promotes the Endocytosis of Transferrin in the African Trypanosome**. *ACS Infect Dis* 2016, **2**(7):518-528.

20. Andre J, Kerry L, Qi X, Hawkins E, Drizyte K, Ginger ML, McKean PG: **An alternative model for the role of RP2 protein in flagellum assembly in the African trypanosome**. *The Journal of biological chemistry* 2014, **289**(1):464-475.

21. Hu H, Liu Y, Zhou Q, Siegel S, Li Z: **The Centriole Cartwheel Protein SAS-6 in Trypanosoma brucei Is Required for Probasal Body Biogenesis and Flagellum Assembly**. *Eukaryotic cell* 2015, **14**(9):898-907.

22. He CY, Pypaert M, Warren G: **Golgi duplication in Trypanosoma brucei requires Centrin2**. *Science* 2005, **310**(5751):1196-1198.

23. Sullenberger C, Pique D, Ogata Y, Mensa-Wilmot K: **AEE788 Inhibits Basal Body Assembly and Blocks DNA Replication in the African Trypanosome**. *Mol Pharmacol* 2017, **91**(5):17.

24. Mann M: **Functional and quantitative proteomics using SILAC**. *Nat Rev Mol Cell Biol* 2006, **7**(12):952-958.

25. Butter F, Bucerius F, Michel M, Cicova Z, Mann M, Janzen CJ: **Comparative proteomics of two life cycle stages of stable isotope-labeled Trypanosoma brucei reveals novel components of the parasite's host adaptation machinery**. *Molecular & cellular proteomics : MCP* 2013, **12**(1):172-179.

26. Guyett PJ, Behera R, Ogata Y, Pollastri M, Mensa-Wilmot K: **Novel Effects of Lapatinib Revealed in the African Trypanosome by Using Hypothesis-Generating Proteomics and Chemical Biology Strategies**. *Antimicrobial agents and chemotherapy* 2017, **61**(2).

27. Domon B, Aebersold R: **Options and considerations when selecting a quantitative proteomics strategy**. *Nat Biotechnol* 2010, **28**(7):710-721.
