# Supplementary material for: Casein kinase TbCK1.2 regulates division of kinetoplast DNA, and movement of basal bodies in the African trypanosome
Source: PLoS One. 2021 Apr 16;16(4):e0249908. doi: 10.1371/journal.pone.0249908 (PMC8051774; doi:10.1371/journal.pone.0249908)
Supplement: Supplementary file 6 [file pone.0249908.s006.docx]

Table S1

kDNA intensity measurements
Supplemental statistics

	- Tet 1K1N	- Tet 2K1N	- Tet 2K2N	+ Tet 1K1N	+ Tet 1K2N
**Number of kinetoplasts**	100	88	84	143	71
**Percent of kinetoplasts above 95% of 1K1N minus-tet kDNA content**	5	0	0	33.6	55.0
**95% confidence interval of the median**	9,224 – 13,233	6,180 – 7,044	6,598 – 7,692	12,874 – 16,532	21,203 – 25,950
