# Supplementary material for: Casein kinase TbCK1.2 regulates division of kinetoplast DNA, and movement of basal bodies in the African trypanosome
Source: PLoS One. 2021 Apr 16;16(4):e0249908. doi: 10.1371/journal.pone.0249908 (PMC8051774; doi:10.1371/journal.pone.0249908)
Supplement: Supplementary file 7 [file pone.0249908.s007.docx]

Table S2

A

	1 Mature Basal Body, mBB-pBB		
	Number of cells	Median inter-basal body distance (nm)	Inter-basal body distance, 5th-95th percentiles (nm)
**DMSO**	41	421	321-597
**AEE788**	131	401	277-535
**1.5 Hr Recovery**	99	377	252-497
**2 Hr Recovery**	106	443	328-563
**3 Hr Recovery**	62	433	301-516

B

	2 Mature Basal Bodies, pBB-pBB		
	Number of cells	Median inter-basal body distance (nm)	Inter-basal body distance, 5th-95th percentiles (nm)
**DMSO**	58	905	428-1425
**AEE788**	29	895	388-1709
**1.5 Hr Recovery**	16	1122	370-1803
**2 Hr Recovery**	32	1015	312-1524
**3 Hr Recovery**	92	844	445-1169

C

	2 Mature Basal Bodies, mBB-mBB		
	Number of cells	Median inter-basal body distance (nm)	Inter-basal body distance, 5th-95th percentiles (nm)
**DMSO**	58	1375	783-1949
**AEE788**	29	1336	611-1922
**1.5 Hr Recovery**	16	1535	683-2306
**2 Hr Recovery**	32	1511	794-2033
**3 Hr Recovery**	92	1303	864-1700
